# The Current Impact of Incidental Findings Found during Neuroimaging on Neurologists’ Workloads

**DOI:** 10.1371/journal.pone.0118155

**Published:** 2015-02-27

**Authors:** Thomas C. Booth, Jennifer M. Boyd-Ellison

**Affiliations:** 1 Lysholm Department of Neuroradiology, National Hospital for Neurology and Neurosurgery, Queen Square, London, United Kingdom; 2 Department of Clinical Neurosciences, Western General Hospital, Edinburgh, United Kingdom; University of New Mexico, UNITED STATES

## Abstract

**Objective:**

Neuroimaging is an important diagnostic tool in the assessment of neurological disease, but often unmasks Incidental Findings (IFs). The negative impacts of IFs, such as ‘patient’ anxiety, present neurologists with management dilemmas, largely due to the limited knowledge base surrounding the medical significance of these IFs. In particular, the lack of evidence-based clinical trials investigating the efficacy of treatments for subclinical IFs makes management protocols challenging. The objective was to determine the impact IFs may have on neurologists’ workloads and healthcare budgets and to examine neurologists’ concerns regarding the clinical management of these ‘patients’.

**Methods:**

Qualitative research based on constructivist grounded theory. Data was collected through semi-structured interviews of purposively sampled neurologists, coded, and concurrent comparative analysis performed. A substantive theory of the ‘IF impacts’ was developed after concept saturation.

**Results:**

Neurologists managed the escalating workload caused by an increased number of referrals of ‘patients’ with IFs found during neuroimaging; however it was unclear whether this was sustainable in the future. Neurologists experienced IF management dilemmas and spent more time with ‘patients’ affected by anxiety. The lack of information provided to those undergoing neuroimaging by the referring clinician regarding the possibility of discovering IFs was highlighted.

**Conclusion:**

The impact of IFs upon the neurologist, ‘patient’ and the health institution appeared considerable. Further research determining the natural history of subclinical IFs and the efficacy of intervention will help to alleviate these issues.

## Introduction

Neuroimaging is vital in the diagnosis of neurological disease and patient management [1]. However, neuroimaging sometimes reveals an IF, of which one definition designed for research, is [2]:

“a finding that has potential health or reproductive importance which is discovered in the course of neuroimaging, but is beyond the aims of the study.”

**Fig 1:**
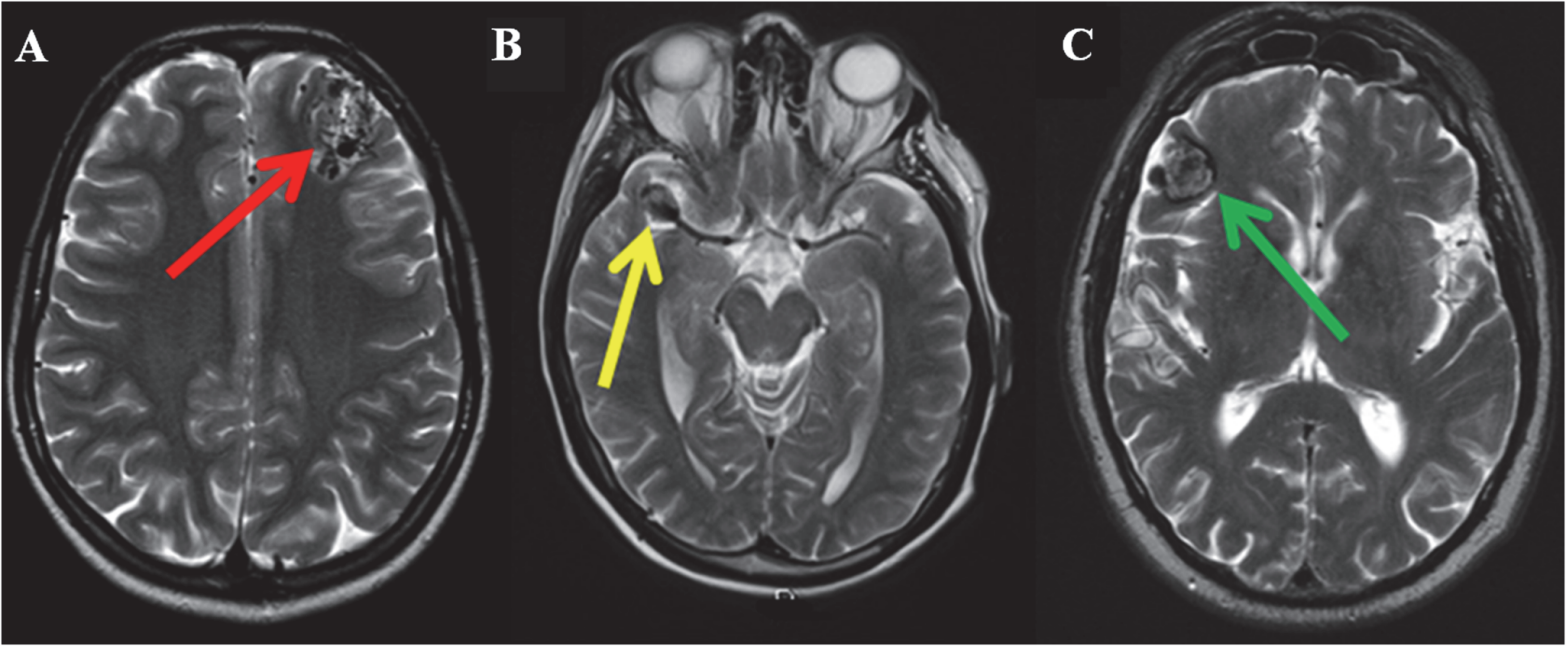
Examples of IFs on axial *T*_2_ weighted sequences: (A) an arteriovenous malformation (AVM) in the left frontal lobe (red arrow); (B) an aneurysm at the right middle cerebral artery bifurcation (yellow arrow); (C) a cavernoma in the right frontal lobe (green arrow).

The increased availability of cross sectional imaging, together with the growing number of imaging techniques and the associated improvement of image resolution has increased the prevalence of IFs [1]. Attempts have been made to establish the prevalence within the general population who appear healthy (i.e. without any signs or symptoms of disease). Using the definition above, neuroimaging IFs are common with an overall prevalence of 5–20% as demonstrated in the only systematic review and meta-analysis [1], although this exceeds 30% in a recent study of an older population [3]. However, the percentage of clinically serious abnormalities is low, ranging from 0.3–3.4% [1,2]. Common neuroimaging IFs considered clinically irrelevant and relevant are listed in Table 1 and three examples are shown in Fig. 1.

**Table 1:** Clinically irrelevant and relevant IFs [1,39–43].

Clinically irrelevant IFs	%	Clinically relevant IFs	%
Cavum septi pellucidi	68*	Neoplastic- benign or malignant tumours	0.7
Enlarged Virchow Robin spaces	51	Colloid cysts	0.04
Absent septum pellucidum	0.002	Inflammatory lesions suggestive of multiple sclerosis	0.04
Ventricular asymmetry	6.1	Hydrocephalus	0.1
Mega cisterna magna	0.25	Cerebral aneurysm	0.35
White matter hyperintensities†	92‡	Arteriovenous malformations (AVM)	0.05
		Cavernoma	0.16

* young adults

†limited in number and not conforming to a disease pattern

‡percentage referring to 65–75 year olds.

IFs and the lack of clarity surrounding their management are not new concepts, for example there was little contemporaneous understanding as to the significance of IFs discovered in a MRI study in 1992 [4]. The challenge continues today as the rapid evolution in image technology limits the experience of neuroradiologists in reviewing novel images. Furthermore, few resources, such as imaging atlases, exist in order to help determine the clinical relevance of an IF [5].

Most unexpected anomalies discovered during imaging are likely to be of little or no clinical relevance but often both the clinician and the newly labelled ‘patient’ [6] want to evaluate them further as both are unwilling to accept the uncertainty that often surrounds the diagnosis and the subsequent natural history of the anomaly [7] (‘patient’ appears in quotations because the anomaly will usually be managed in the healthcare system until it is explicitly determined to be of no clinical relevance without need for medical follow up). Evaluating an IF may subject the ‘patient’ to needless testing, and in some cases needless treatments, which on occasion may be inconclusive or harmful [1]. Contrary to some lay views, early intervention for a clinically serious or life threatening IF does not always equate to a successful outcome. It may only mean that the patient discovers an illness at an earlier date [8].

‘Patient’ anxiety can be a serious consequence of discovering IFs. ‘Patients’ with IFs might also incur financial penalties due to the working days lost whilst undergoing subsequent investigations and medical consultations [6,8], and the transport costs to and from hospital [1]. Financially worse still, uncovering an IF might invalidate future life and health insurance [1,4,6].

In the UK, the National Health Service (NHS) will usually pay for additional diagnostic investigations to characterise or follow-up an IF but in other healthcare systems medical insurance institutions might need to absorb the costs. Another potential institutional cost may be in the form of litigation if ‘patients’ with an IF are not notified [9]. Therefore, costs from IFs include those to the institutions administering healthcare as well as ‘patients’.

Conflicting advice remains regarding the optimal management of ‘patients’ with IFs [1,10–14]. In summary, clinicians, in particular the neurologist to whom many ‘patients’ with IFs are referred, appear to have an increased workload due to the increased prevalence of IFs and experience dilemmas regarding how to manage IFs discovered with neuroimaging.

The aim of this study was to determine neurologists’ current concerns regarding the impact that IFs have on their workloads and healthcare budgets, as well as to examine their concerns regarding the clinical management of these ‘patients’.

## Materials and Methods

Written confirmation that the study did not require Research Ethics Committee approval was provided by the South East Scotland Research Ethics Service, UK.

The current lack of research investigating the neurologists’ experiences dealing with IFs led to the identification of Grounded Theory (GT) as the prospective qualitative research method of choice for this study. The Constructivist GT (CGT) approach developed by Charmaz [15] was preferred as opposed to the considered conventional GT [16,17]. The CGT approach acknowledges the researcher will have been influenced in some manner by their own experiences [18], as is the case in this study where the authors had insight into the referral patterns of patients with IFs discovered during neuroimaging. CGT incorporates the use of note taking and Constant Comparative Analysis which compares one data set continually with the next. All participant interview transcripts were analysed with this method to ensure that all data extracted was the participant’s alone [16].

Purposive sampling targeted a small group [19] of neurologists working within a neurosciences unit. This subject group are referred ‘patients’ with IFs from General Practitioners (GPs) and other hospital specialists. Senior neurologists (UK consultant and specialist registrar grade) currently employed at NHS Lothian Department of Clinical Neurosciences, UK, were emailed individually with an invitation to join the study and were given a Participant Information Form.

A consent form was completed by both the researcher and participant. Participants gave permission to have their interviews recorded in accordance with GT principles [20], which were transcribed verbatim using Constant Comparative Analysis before the next participant was interviewed. This enabled relevant themes that emerged during the interview to be pursued in the next interview [20,21]. To further improve validity participants were questioned about specific concerns they may have regarding IFs (Appendix 1). The words and sentences in the transcribed text were repeatedly examined to provide provisional code related to the research question. A search for patterns in the provisional code allow the data to be reduced into groups of different and similar focused codes [22,23]. Further refinement of focused code formed theoretical categories [22,24]. Appendix 2 shows how the data were refined using CGT. New participants were brought into the study until data became saturated and no new themes emerged which is how the methodology achieves acceptable reliability and validity [15,21,25]. All data was collected and analysed by one researcher (S1 Appendix, S2 Appendix).

## Results

### GT interview results

Eight senior neurologists were enrolled in the study (six consultants and two specialist registrars), from a total of 12 approached in 2012. Of the four who did not enrol, three were consultants and one a specialist registrar. Three did not reply to the request to join the study and one did not consult patients found with IFs. The analysis of interview transcripts (S3 Appendix) elicited the core category title ‘incidental phenomenon’ which included IFs as described above in addition to other findings that do not have potential health or reproductive importance. Four theoretical categories emerged from the focused coding each relating to the core category which were Challenges for Patients and Clinicians Regarding Treatment and Management; The Increased Role of the Radiology Department; Innovations to the Participants’ Practice; and Financial Challenges. No perceptible differences in responses were seen between consultants and specialist registrars, except regards comments on private practice (consultants alone discussed this) and training (specialist registrars alone discussed this), otherwise the numbers were too small for a meaningful analysis.

### Challenges for patients and clinicians regarding treatment and management

Participants offered a consistent view that, despite a large number of referrals to see ‘patients’ with IFs, they were able to manage their overall workload. However, they feared that this will change in the future. Participants stated that ‘patient’ anxiety needed to be addressed before discussions could begin regarding treatment choices and was associated with increased workload due to lengthy or additional consultations:

“They can crescendo into an acute anxiety state by the time they come to see the neurologist. They have convinced themselves they have MS [multiple sclerosis] and you then have to try and dismantle that, and, it is not going to take 10 mins.”

Most referrals emanated from Ear, Nose and Throat specialists and GPs. A significant problem that three participants encountered occurred when ‘patients’ that had been referred to them from other specialities had been given incorrect information about the IF which was sometimes due to poor communication and was often associated with ‘patient’ anxiety:

“In fact, the irony is the patients always seem to take the word of a doctor not specialised in neurology, which can be very frustrating”.

There was disagreement between neurologists regarding the definition, significance and optimal management of IFs reflecting the published literature [1,2,26]. This was compounded by the non-specific nature with which IFs can be reported by radiologists, especially non-neuroradiologists:

“if they [radiologists] don’t know what it is, again you are going to have to follow it up with surveillance so it’s not a treatment dilemma but a management dilemma”

The disagreement regarding the significance and optimal management of IFs was clinically relevant: “I think at the worst end of the spectrum it can lead to inappropriate diagnosis and even inappropriate intervention”. An account was described where ‘patients’ had decompressive surgery for Chiari I malformations when, in the opinion of one participant, the anomaly should remain under surveillance. Indeed, the optimal treatment for some IFs was found to be particularly problematic, for example cerebral aneurysms and AVMs (Arterial Venous Malformations) due to the current limited evidence-base to guide clinical decision making. Similarly the management of enlarged Vichow Robin spaces was problematic:

“I had previously disregarded them as normal, simply incidental, but now looking at research maybe they are not; maybe I should be worried about this ‘patient’”.

White matter changes also provided a management dilemma:

“Should those ‘patients’ be treated in the same way as someone who has presented with a transient ischemic attack? Should they be started on an anti-platelet drug? A lot of people do start them on an anti-platelet drug. I have to say, I don’t because there is no evidence to support it and there is no evidence not to support it”

All participants expressed concern that they may be wrongly managing their ‘patients’: “we really don’t know if they [IFs] are going to influence health in the long term. It is very difficult to give clear advice.” To accumulate more knowledge on the long term effects of IFs, attempts by some participants were made to follow-up ‘patients’ after a consultation. There was agreement that further research is required to assess the potential risks of these IFs and more evidence-based clinical trials are required to demonstrate efficacy of interventions.

Litigation initiated by ‘patients’ who had not been informed of an IF, was also discussed. Participants stated that every ‘patient’ should be informed of all newly discovered IFs otherwise there would be repercussions for the participants themselves:

“There is an obligation to be completely open with the ‘patient’. You might well get into trouble if you identify an IF and don’t tell the ‘patient’ about it”.

Despite the neurologists’ direct advice against further imaging in certain scenarios, often the ‘patient’ or the ‘patient’s’ family felt that further unnecessary diagnostic investigations (especially further imaging) were required to characterise the IF. Neurologists felt unanimously under pressure to agree to this. One participant felt these consultations were difficult because ‘patients’ believed that “the doctor is your servant, no-longer the guide”. The six consultants found it easier to reassure a NHS patient that they did not require further tests or imaging however, private patients’ attitudes were different and they were paraphrased as saying “well I’ve paid my premiums I am entitled to my scan”.

A lay awareness of the risks from radiation exposure and the perception that MRI is the ‘gold standard’ in neuroimaging meant that ‘patients’ specifically insisted upon further characterization with MRI. Participants agreed that the more anxious the ‘patient’, the more likely that they would want further unnecessary invasive diagnostic procedures and treatment despite the associated procedural risks.

### The increased role of the radiology department

It was claimed that with the increased availability and access to neuroimaging, patients are being unnecessarily over-investigated with the ‘patient’ in some cases driving the demand. It was stated that:

“its mere availability means it gets used more; statistically it means you know more of these things [IFs] will be discovered”.

Furthermore:

“I think this is the way modern medicine has gone; if you over-investigate people you will uncover more, the VOMIT theory (Victim Of Medical Imaging Technology)” [9,27].

It was noted that there was an increase in ‘patients’ with IFs referred to neurology from radiology, then referred back to radiology for further imaging to characterise the IF or determine its management since the initial imaging sequences had not been optimised for that anomaly. Participants claimed technological advances in imaging techniques and image resolution improvements were uncovering more unexpected anomalies and that characterising such anomalies was increasingly difficult:

“The MRI scans are getting so sensitive they are picking things up earlier and earlier in the disease process, we are still learning what the significance of these finding are.”

Furthermore:

“[images are being] scrutinised by the radiologists having acquired them in fine detail and then reformatting them, maybe spending longer viewing them “.

Participants frequently felt that the best course of action for these ‘patients’ was to place them under observation and refer them for follow-up imaging. There appeared to be no difference between consultants and specialist registrars in the management of IFs.

If an aneurysm or AVM is found “then quite often MRI or catheter angiography may be appropriate especially when it comes to putting patient into low, medium or high risk groups with respect to their future prognosis, then the decision whether or not to treat them follows and if the decision to treat is made, then there is a whole host of further investigations and expensive treatment [usually in the Radiology Department] and follow up, that follow”.

An area of rising concern was the potential for considerable workload impacts as the current UK population is expected to live longer. Participants cited that current research has stated that increasing age is congruent with the increase in IFs [1]. More elderly patients were being referred for neuroimaging and, when an IF was unmasked, more were being referred to neurology then radiology for characterisation in the cyclical manner described above.

### Innovations to the participants’ practice

Participants altered their working practice to accommodate the IFs. Participants informed patients who had no neurological clinical features and who insisted that they undergo neuroimaging, about IFs and their associated risks (as has been recommended for research study volunteers [28]):

“What you should do before any test is to explain the possible consequences of that test and any potential adverse consequences of those tests. So we should be having these conversations [about IFs and their associated risks] with these patients anyway.”

Furthermore:

“. … I explained. ….there is a 1 in 20 chance we may find an IF, of a cyst or some white blobs or, something that has probably been there since you were born that is not the cause of your symptoms so, I think anticipating them helps when they do crop up”

Many neurologists used the phrase “opening a can of worms” or “playing roulette” to explain this to their ‘patients’:

“We should warn them of IFs before the scans, explain there is a 1 in 37 chance of finding something on an MRI basically they are playing roulette having an MRI. I try to impress upon patients who have headaches and who are determined to have a scan that’s the risk they are taking undertaking an MRI.”

Some ‘patients’ claimed that they were informed of their IF inappropriately by clinicians who were not neurologists, either by letter or through a consultation with their GP. This prompted some participants to provide ‘patients’ with information leaflets about their conditions before their consultations in an attempt to reduce anxiety. Some participants thought that an “IF help desk” should be implemented to provide advice for concerned clinicians. In many circumstances the ‘patient’ would then be told by their clinician that they need not be alarmed, forgoing the anxious wait for a neurology consultation.

Multidisciplinary neuroradiology meetings and interventional neuroradiology clinics, where IFs were discussed, were found to be vital in the management of these ‘patients’.

Both specialist registrars interviewed had also been trained to think carefully before requesting imaging:

“As I have gone through my training I think long and hard before I request a scan for which I do not have any clear justification and that’s chiefly done because, it is good clinical practice and partly because, I do not wish to uncover any IF and [partly] not knowing what to do with half of the IFs”.

### Financial challenges

One neurologist commented that “the costs for the NHS [in managing IFs] just wrack up”. Another said:

“There are costs in terms of our time talking with the patients allaying their fears which takes a huge amount of effort, researching the finding, research any evidence-based managements, going to meetings to discuss the abnormality and the costs of the further testing, further imaging, further consultations”

As discussed earlier, participants claimed consultations with anxious ‘patients’ took longer than an average appointment and on occasion required a second consultation. Participants stated that further imaging was often requested either for characterisation or surveillance of IFs. Half the participants stated that this was done frequently with further imaging required 50–75% of the time, whereas the other half described this as occasional. The participants spoke of costs for surveillance imaging of IFs e.g. meningiomas, Chiari 1 malformations and small cerebral aneurysms, as well as imaging for IF characterisation e.g. possible cavernomas or non-specific high signal intensity lesions. Where indicated, the high cost of cerebral aneurysm and AVM referral (initial multidisciplinary meetings and neurovascular clinics), treatment (interventional neuroradiology, neurosurgery or stereotactic radiosurgery) and subsequent management (inpatient stay in some cases, including a High Dependency Unit bed) was highlighted.

For illustrative purposes we have estimated some of the costs in the studied healthcare system using the NHS Information Services Division (ISD) Cost Book 2012 [29] (Table 2). The initial referral for a neurology consultation of a patient found with an IF cost £ 256 (US$ 430). If the ‘patient’ was not discharged at this point but referred for an MRI scan to characterise the IF, then there was a further cost of £ 223 (US$ 374) which was associated with a follow-up neurology consultation costing £ 256 (US$ 430).

**Table 2:** Costs of IF investigation and treatment.

Procedure	Source	Costs (US $)
**Neurology consultation**	ISD*	430
**Brain CT scan**	ISD	201
**Brain MRI scan**	ISD	374
**Catheter cerebral angiogram**	Lothian private price list	1642
**Cerebral embolisation**	Unpac 2010–2011	4480†
**High Dependency Unit bed** ‡	ISD	1178

* Information Services Division Cost Book 27/11/2012

† The NHS does not have a fixed cerebral aneurysm embolisation tariff for procedures within the catchment studied. The cost quoted is the staffing cost and did not include consumables.

‡ per day cost (post embolisation)

Participants requested MRI in the first instance over the cheaper option of computed tomography (CT) (£ 223 (US$ 374) compared to £ 120 (US$ 201)). This was mainly due to ‘patient’ preference, including refusal to undergo CT scanning, but neurologists also claimed futility of CT scanning as ‘patients’ invariably insisted on a MRI scan after a normal CT report.

Participants also described the financial penalty to ‘patients’ with IFs due to travel costs to clinics, overnight accommodation should they live a distance from the hospital, and time off from work—the latter impacting on their income if they do not have paid sickness absence or are self-employed. Furthermore, it was noted that following the discovery of an IF, insurance companies would increase the insurance premiums of ‘patients’, even if the IF was considered clinically irrelevant. In some cases IFs impacted heavily on the ‘patient’s’ career. For example, ‘patients’ who were heavy goods vehicle and public service vehicle licence holders (UK Driver and Vehicle Licensing Agency Group 2) were unable to drive following the discovery of an aneurysm, however small. One neurologist summarised such a problem:

“So their own costs go up and their income goes down because they are, for example, taking time off work; these are hidden costs I think as well to IF”.

## Discussion

### Implication of this Study and Comparison to Other Studies

Participants stated that they were managing an increased IF workload. However, participants expected that an ageing population, along with the increased availability and technological advances of imaging, would bring renewed demands upon their future services. It was noted that the views of the participants in the current study are concordant with other authors who show that one reason for an increased workload is that ‘patients’ insist that they undergo imaging [1].

As described in other studies, concerns were highlighted that IFs often cause patient anxiety due to the uncertainties surrounding their clinical relevance and their natural history and that other detrimental effects might include difficulty obtaining health or life insurance [6,8,28]. The lack of evidence-based clinical treatments of IFs made management decisions challenging, prompting a review of these ‘patients’ in multidisciplinary neuroradiology meetings and neuro-interventional clinics. Nonetheless, it is noted that an IF might be of unequivocal benefit to the patient if the lesion is treatable and early diagnosis improves outcome [30], for example the detection of a > 25 mm posterior circulation cerebral aneurysm in a young patient [31].

The results also concur with other reports that clinicians in general are becoming increasingly reliant upon further imaging to characterise IFs [1] and reduce the chance of possible litigation [28]. This was compounded by the non-specific nature with which IFs can be reported by radiologists, in particular by non-neuroradiologists as seen in other studies [1]. There was agreement with other studies that further imaging to fully characterise an IF and subsequent treatment can carry risks [1,5,6], for example, a cerebral aneurysm may require an angiogram. Sometimes no medical intervention is indicated but nonetheless a ‘healthy’ individual might become labelled as a ‘patient’ [6].

IFs were demonstrated to result in financial costs concordant with previous studies. For example, a Japanese neuroimaging study found that if brain MRI is used to screen healthy individuals for significant pathology, at US $200 per MRI scan, it would cost $24,733 for each significant abnormality identified [32]. Cross-sectional body imaging studies have also demonstrated financial penalties. A CT angiogram study performed in the US estimated that the total cost of investigating the extra-renal IFs of 49 patients was US$ 6137 [33] and a UK body imaging study showed that total cost of investigating IFs of 29 patients was US$ 12775 [34].

Neuroimaging research has shown that IFs are common and problematic [1,6] and in the UK and overseas the management of IFs varies between research centres [8,28,35]. This study found that participants in a UK non-research setting also recognised that IFs were problematic and that the management of IFs varies [34]. For example, some participants did not discuss IFs with patients before imaging whereas others discussed with their ‘patients’ the possibility of discovering IFs before any imaging. Some only discussed IFs when their ‘patients’ insisted on neuroimaging that was not clinically indicated. The informative approaches made ‘patients’ aware of the issues surrounding IFs before they gave their consent for imaging. Such approaches appear to have optimised the participants’ clinical practice according to UK government guidelines [36].

The UK imaging research community have recently held discussions regarding the management of volunteers undergoing research involving imaging. The consensually obtained guidance states that informed consent should be standardised and implemented for all research volunteers before they can enter studies [6]. This guidance aims to educate the imaging research community, and ultimately volunteers, to ensure all are fully aware of IFs and their impacts. We found no literature or clinical guidelines that recommended clinical patients, as opposed to healthy volunteers, should be apprised of the existence of IFs prior to imaging. Although inappropriate in many scenarios, for example neuroimaging in an emergency, in other scenarios information on IFs prior to imaging would enable patients to actively participate in their own healthcare, thereby maintaining good ethical conduct [37,38].

### Limitations and Strengths of Study

Although the interviewing was appropriately semi-structured without *a priori* pre-conceptions or constraints there is the potential for heterogeneous data to make categorisation difficult. Although data saturation was achieved with a small sample size, which is appropriate in interview-based qualitative research [38], the potential for sampling error is large which limits the generalizability of the results. Despite these limitations, the themes reported by the participants recurred independently during interview, which suggests that these results reflect the opinions of those neurologists who are referred ‘patients’ with IFs in the catchment studied.

### Future Directions

Interviewing neurologists from other UK neurology departments might increase the generalizability of the findings found in this study. Further understanding of the impact of IFs found during neuroimaging on neurologists’ workloads might also be achieved with the implementation of a larger National survey designed using the findings of the current study.

This study and other studies also show that further research is needed to characterise and determine the natural history of IFs, especially new phenomena found using high resolution or advanced imaging. More clinical trials are necessary to understand the efficacy of treatments for patients with subclinical IFs in order to provide guidelines for clinicians to effectively manage their patients. Research looking into patients’ attitudes towards IFs and how to reduce unnecessary anxiety would also be valuable.

## Conclusions

Neuroimaging IFs continue to impact on neurologists, ‘patients’ and the parent healthcare institution. If ‘patients’ need to be cautious of VOMIT [27], perhaps the healthcare system needs to be cautious of SPEW (Scans Propagating Exponential Workloads). Management approaches have been described in the research community as well as in the clinical arena as described in this study. Issues surrounding consent and disclosure of IFs are common to both research and clinical imaging, however determining the indications for imaging in the first instance is a singularly clinical management issue. Participants felt that clinical patients should be informed of the risks of IFs prior to imaging in the same way that research volunteers are, especially when patients insist that they undergo neuroimaging. This study suggests that, as a minimum standard of care and when clinically appropriate, informed consent prior to neuroimaging might be prudent.

## Supporting Information

S1 Appendix Participants were asked the following questions if they did not address them during the interview. (DOCX)

S2 Appendix Progression from provisional codes to theoretical categories. (DOCX)

S3 Appendix Interview transcripts. Interview transcripts from eight participants.
(DOC)
