# Supplementary material for: The Current Impact of Incidental Findings Found during Neuroimaging on Neurologists’ Workloads
Source: PLoS One. 2015 Feb 27;10(2):e0118155. doi: 10.1371/journal.pone.0118155 (PMC4344225; doi:10.1371/journal.pone.0118155)
Supplement: S1 Appendix — (DOCX) [file pone.0118155.s001.docx]

**S1 Appendix.** Participants were asked the following questions if they did not address them during the interview.

| Questions |
| --- |
| IFs found within the brain are currently the subject of much academic press at the moment, with patients being referred from clinical, research and private practice. I was wondering what your views were upon the topic.  Could you describe how IFs have impacted upon your workload.  How much of your time do you feel ‘patients’ with IF take up?  Could you describe how ‘patients’ with IFs present to you.  In your experience how often do you feel the necessity to request further imaging or intervention [for ‘patients’ with IFs]?  Must you do extra blood tests or anything else [for ‘patients’ with IFs]?  Could you describe the impacts IFs have upon the ‘patient’.  Have you ever felt pressurised by a ‘patient’ to request a scan?  In your clinical practice do you explain the risks of finding an IF to patients?  In your experience have you found any issues regarding managing ‘patients’ found with IFs?  Have you noticed any subtle differences in the number or type of IFs due to the advances in imaging technology, for example scanners with higher field strength?  Do you hold any opinions and views regarding the management of ‘patients’ with IFs?  Have you anything else you wish to add surrounding the topic of IFs? |
