# Supplementary material for: The Current Impact of Incidental Findings Found during Neuroimaging on Neurologists’ Workloads
Source: PLoS One. 2015 Feb 27;10(2):e0118155. doi: 10.1371/journal.pone.0118155 (PMC4344225; doi:10.1371/journal.pone.0118155)
Supplement: S2 Appendix — (DOCX) [file pone.0118155.s002.docx]

**S2 Appendix.** Progression from provisional codes to theoretical categories.

| Provisional codes | Focused codes | Theoretical categories |
| --- | --- | --- |
| Patient anxiety/confusion  Patient fear of unknown  Patient costs  Uncertainty for the patient  Treatment concerns for the patient  Life insurance issues  Employment issues | Effects on patients from IFs | Challenges for Patients and Clinicians Regarding Treatment and Management |
| Patients have many questions  Angry patients  Lengthy consultation times  Challenging consultations | Effects upon neurologists from patients found with IF |  |
| Further imaging to clarify IF  Further consultations with neurology regarding IFs  Challenging decisions for patients regarding treatments | Treatment and management challenges for patients found with asymptomatic IFs |  |
| Longer waiting lists for symptomatic patients  Patients insist upon further imaging  Patients insist upon MRI over CT  Patients do not listen to neurology  Possible legal implications  Further consultations with neurology regarding IFs | Treatment and management challenges for neurologists with patients found with asymptomatic IFs |  |
| Increased scanning  Increased technology  Favoured move towards MRI  Patients insist upon further imaging  Patients insist upon MRI over CT | Lack of information regarding IF leading to onward referral/tests | The Increased Role of the Radiology Department |
| Multidisciplinary meeting  Informing patients pressing for imaging of IF  Help desk to reduce impact upon neurology regarding IFs  Different preparation for consultations of IF  Leaflets on IFs  Informed consent | Methods of reducing impending workload and financial impact of IFs upon neurologists | Innovations to the Participants’ Practice |
| Lack of evidence-based treatments for asymptomatic findings  Further research for management  Further research for treatment | Further research upon asymptomatic IF their aetiology and management |  |
| Unnecessary imaging finding IFs  VOMIT theory  NHS costs potentially escalating  Patient costs  Life insurance issues  Employment issues  Further consultations with neurology regarding IFs  Favoured move towards MRI | Impact of Increasing scanners numbers and technology uncovering IFs | Financial Challenges |
