# Supplementary material for: The Current Impact of Incidental Findings Found during Neuroimaging on Neurologists’ Workloads
Source: PLoS One. 2015 Feb 27;10(2):e0118155. doi: 10.1371/journal.pone.0118155 (PMC4344225; doi:10.1371/journal.pone.0118155)
Supplement: S3 Appendix — Interview transcripts from eight participants. (DOC) [file pone.0118155.s003.doc]

**Appendix S1: Interview transcripts.** Interview transcripts from eight participants.

Participant 1

I - interviewer

P - participant

I: IFs found within the brain are currently holding a lot of academic press at the moment, with patients being referred from clinical, research and private practice. I was wondering what your views were upon the topic.

P: I haven’t seen any publications about it in the lay press but, personally I am aware of the publications in scientific journals and guide-lines and I’m generally aware of it as an issue. I didn’t have an MRI in a research scanner until I had life insurance; my wife too got life insurance before she had a scan. So yes, I’m very aware of incidental findings and I personally wouldn’t be in a research study unless I had life insurance. If I was planning to have a mortgage I wouldn’t be in a research study.

I: How do you explain to a person wanting to have a scan what sort of problems there may be?

P: Clinically? Or, only in research?

I: Both, but also commercial.

P: I think when you are referring anyone for a scan you have to be aware that you might find something and also you may not find what you are looking for. There are less life insurance implications when you are referring someone for a clinical scan because as soon as there is indication for a scan then life insurance implications will kick. You can’t get away from the fact that if you have signs and symptoms of a brain tumour and you have it documented in your medical notes. Then you will struggle to get insurance, even before your scan because the insurer will take it more seriously. In clinical practice, if you are ordering a scan for a reason you shouldn’t be, or if you order a scan for not a particularly good reason and I think the issue does comes up in clinical practice. When you have a very anxious patient who is desperate for scan to explain symptoms they have, that may not be even that severe but, they want assurance do you give in and give a scan anyway, if you think it will reassure them, yes I do. But if you find an IF that is of real no significance then they fixate on it, and believe it is an entire explanation for their symptoms which may have been totally unrelated.

I: Have you ever felt pressurised by a patient to request a scan?

P: I think there are a reasonable number of patients who are particularly keen to have the best scan they can get. My practice yes, certain cultures, a lot of emphasis is placed upon the scan, certainly South Asian culture patients are rarely satisfied with a clinical diagnosis and in Eastern European culture Americans, again you do feel under pressure they do not believe the clinical diagnosis without a scan.

I: Do you find this puts you under a lot of additional workload when……

*Clinician cuts in.*

P: Yes, yes, they are a lot of work anyway, you have to put the time in to give them reassurance and sometimes it is appropriate to do a scan. For some patients it would solve the problem if their mother had a brain tumour and they have a minor headache they think they have a brain tumour. You may well be quite sympathetic and think you might as well get a scan to give then peace of mind and rule out a brain tumour but, then you ask yourself why did I give and agree to the scan. I personally just try and do routine investigations unless I know the result will affect my practice and I often talk to patients and ask do want you want to investigate this further? How far do you want to go? And most people are sensible but if people are very anxious it can be very difficult. You do your best to reassure then but sometimes you do have to give in and give them a scan to move the doctor- patient relationship onwards. It might well be they are simply not going to believe you without a scan and if you have got to convince them that their symptoms are functional then you may not be able to do this fully until they have the results of an MRI scan.

I: Do you find any other additional costs for example blood tests or anything else?

P: Yes, the costs of the investigation and the findings in general often in a specific situation to reassure someone who is very anxious can be expensive and time consuming, it can often save money doing a scan because you may have to see them in clinic 4 or 5 times to reassure them properly because clinic appointments are expensive. An out-patient appointment is about £150 I think I’m not sure if that’s the up to date rate but, we are talking a lot of money so a clinic appointment is in the same region of expense as a CT scan from what I understand so if you are saving yourself eight clinic appointments then you are may well be justified in requesting the scan, but you do try to avoid it. It’s the same with blood tests you shouldn’t do tests you don’t want the answer to.

I: Could you describe how IF have impacted upon your workload

P: Currently the number in my practice is increasing but still manageable. But, when I worked in the North West we had a number of patients who had whole body CT Scan with Life scan. I personally wish to throttle Life scan on a regular basis. If you are an anxious person you will opt to have a scan in the first place that’s the first thing. These people who pay for their scan are those who cannot take a rational explanation or pragmatic approach to an IF. I’ve known people have investigations based on things that have come up from Life Scan that otherwise would have been left. For example, they have had a sigmoidoscopy and other invasive tests that have potential side effects because of an IF found on a Life Scan. Realistically there may have been a chance the result may have been positive but I don’t believe there is any evidence theses services have extended lives or, even benefit anyone I think they are harmful and I am quite angry they exist.

P: In your experience have you found any issues with managing patients found with IFs.

P: Yes, small vessel disease is a notorious one, there is no evidence of benefiting patients by managing the scan in my patient age group we do find small vessel disease but not found from the scan. We do find small vessel disease a lot and we don’t have a proper evidence based way of managing it and you do feel obliged to do something and usually that will involve managing their cardiac risk factors. If they have not had a stroke and there is no indication to do that. You are managing them out-with any evidence based treatments potentially exposing them to harm. It may benefit them but we don’t know that.

I: Can you foresee a problem in the future with the advent of increased scanning impacting on you

P: Oh it’s already a problem but like I said I’m managing but it’s going to get worse, the problem is societies lack of faith with clinical skills that’s what is driving this increased volume of scanning and doctors have lost confidence in their clinical skills. Patients have lost confidence in the diagnosis made by the doctor without a scan. Combine both and that doctors are requesting scans that aren’t necessarily indicated and patients are demanding them yes it will get worse. I think the only solution to this is proper evidence based guidelines when it is and isn’t indicated and doing our best to adhere to them but, guidelines that are not evidence based are no use what so ever because you can find a way around them. Why would I believe them if there is no evidence to support them.

I: Do you feel patients with IF can be managed better or differently?

P: I don’t, I think we are stuck with them. I think the only way to make it better is to not find them and there does need to be regulation about pointless scanning. Certainly commercial screening CT they are a license to intervention with undue harm and I don’t see why current legislation can be used to prevent that. Otherwise it’s not something I can see being solved. In terms of consenting patients for studies I think you would need to have information sheets explaining you may find an IF… it may be severe…. it may cause anxieties …..it may not benefit you to find it. That included in an information sheet would be beneficial it would maybe reduce some of the harm done. For clinical patients with a justified request no, I do not always discuss IFs.

I: When you have patients come to you and they are anxious have you an idea of how much time they can take

P: 45 mins easily, it takes longer than an average appointment the more educated the patient they have more questions to ask yes you can spend twice – three times as long with anxious patient who has got an IF. That can be down to personality sometimes you can tell a patient quite honestly of an IF and it can be dealt with in 10 minutes others longer. Some people respond to better explaining this is an IF do not worry about it, it may have been there all your life, it probably won’t course any harm for the rest of your life, most people are fine with that …the majority are… but, in my experience people referred from commercial scanning aren’t that way inclined. Also people who have research studies are having them specifically for the same reasons as they want a scan to reassure them. I have told someone who I made a diagnosis on the basis of history, it’s a harmless condition and I don’t see any indication for scanning. 2 days later I happen upon them and the person was being scanned as a private patient. I felt awful if they were that worried I would have organised a CT scan. There would have been dubious need for a CT however, and definitely no need for a MRI scan, other than the fact of saving a dose of radiation. I had spent 1 hr 30 mins already on that patient and had they had left appearing to be reassured… I was embarrassed…. some people are so fixated on wanting a scan.

I: In your experience how often do you find the necessity you request further imaging or intervention?

P: I do request further imaging yes, how often ummm mainly because the imaging sequences done have not been appropriate for the IF found so I’ll order another to clarify its aetiology or if something is deemed non-specific I may get further imaging in 6 mths or a yr to keep an eye on it.

I: Have you noticed any subtle differences in imaging resulting in further questions due to the advances in imaging technology and higher field strength scanners?

P: The fine slices and detailed images make us look harder and longer at the imaging simply because there is more to look at, this I believe is making us find more subtle changes, that previously I may have missed leaving me with more questions…was that there before?

I: Papers suggest the older you are the more likely you are to find an IF what have you found in your experience?

P: Certainly older people have different personalities they are a different generation, older people do have more IF. If you find an inoperable brain tumour are you doing them any good apart from organising their palliative care and giving them a prognosis you have caused them a lot of distress.

I: Do you feel the uncovering of IF may affect patients’ Quality of Life?

P: Paternalistically and realistically yes, but having seen patient dying very quickly from a previous unknown the diagnosis of terminal cancer you have given them a chance to put their affairs in order, make a will, say good bye to their families. So I think it can be beneficial but, that is a bit of a difficult question. I will be quite frank with them and ask if they want further scanning most people say no they want to take life as it is.

I: You say paternalistically is that to say you feel as a doctor you have to tell them everything?

P: Depends upon the situation and the personality of the patient what they want to know and the questions they ask, IF can open a can of worms. I say paternalistic in that we may have done you no good by telling you about your IF, but if your IF is a potentially fatal condition and can’t be treated some people feel they have been “done good” by being given a diagnosis. You as a Doctor must think about that before you fill out the imaging request.

I: Have you anything else you wish to add surrounding the topic of IFs?

P: I think screening programmes need to be evaluated fully before they are implemented. Research with evidence based treatments for small vessel disease would help, managing these patients will then become less of a bloody nightmare because we will actually know what to do for the patient.

Participant 2

I: IFs found within the brain are currently holding a lot of academic press at the moment, with patients being referred from clinical, research and private practice. I was wondering what your views were upon the topic.

P: The risk for young people having IFs are relatively low…1 in 200 something like thatish, probably a bit higher if you take into consideration structural issues which can count as IF such as Chairi malformations but…so IF in young people aren’t really that much of a problem for me in my practice because, generally speaking my patient group is substantially older. In the older age group there is a much higher risk of quasi IF those that cause most gip are unruptured aneurysms, which can cause a lot of fuss an awful lot of fuss and quite a lot of harm. Small vessel vascular disease and whether or not it is demyelination disease or not. It’s never demyelination always small vessel disease. Does small vessel disease in a 53 yr old smoker mean something, is it incidental, is it related to something or not, eh I suspect many more of these are incidental.

I: In your experience have you found any issues with managing patients found with IFs especially as you have mentioned unruptured aneurysm and small vessel disease?

P: Management in the broader sense, in that we have the management of the patient. You don’t have a stroke but you do have a 3mm aneurysm in a Cerebellar artery and, we are probably not going to do anything very much because the risk of rupturing is bigger, if they’re larger. But actually the ones that burst are small ones just like the one you’ve got. But due to the risk we aren’t going to do anything about it but it may burst at some point. Pick the patient up of the floor ummm, that is a very difficult patient management conversation to attempt to convey to the patient that they do have a real but small risk of something bad happening to them which they had before and didn’t know about it. And it is a real small risk that is potentially modifiable but the modifiable bit has risks that might make you more crook. So what I am saying is you have an aneurysm but, to treat it might be more dangerous than leaving it. So that’s a really difficult conversation but, it is much more difficult for the patient how do you deal with that information …what do you do… even the most stoic of patients sensible straight forward people get terribly distressed by the whole thing and worry and ruminate about it ..it can be very difficult.. Small vessel vascular disease is another issue. Do I think this is important enough to have to do some kind of intervention to look into the stroke risk in more detail, if this is stroke related and is small vessel disease and, if it is clinically significant and has anything to do with the episode that brought them here ehh and if it does, should I be doing anything about it. I mean if it’s a stroke you should maybe assess them for cholesterol levels.

So again you’re probably going to say to the patient there is something there but, don’t worry about it. And so while people worry about Chairi malformation and AVM’s and low grade gliomas and that sort of thing and, they worry when they happen actually much more commonly in neurology practice are the pseudo IF in the elderly population and, what we are seeing does represent the aging process but, it’s just whether it’s clinically important or not and… err the quality of some of the x-ray reporting is limited err, because so to say this patient has small vessel disease which is probably advanced for age further correlation is recommended rubbish.

I: Do you think the report should go into more detail is there any way to improve it.

P: Well, there are grading for small vessel disease and it would be helpful if they potentially used those. It would also be helpful if the radiologists who have very good radar would call if the scan was normal or not. Some radiologists are very experienced in reporting stroke and their reports are very reliable but, sometimes you get the feeling that the radiologists are putting their fingers in the air and waving it about particularly non-neuroradiologists in some districts were I receive reports from you, just get “advanced for age”… it’s almost like they are frightened to write normal for age …like if they write normal for age and then the patient has a stroke ooh! So they write advanced for age …it’s like they are passing the management buck in a way …..a good study a 1000 normal for age scans reported by neuro radiologist and send them to general radiologists and see if they were reported a slightly advanced for age because they might well be.

I: Would it help if the radiology reports were much more specific

P: Measure it and then you can do research so, if you have a way of saying this is grade one, two…three...four.... Whatever. And then someone can do the epidemiology. If you are 50 and you have grade 1 disease and nothing happens, if you have grade 2 disease and you are treated with statins ……..if you have a very loose and imprecise radiology report which says advanced for age it is then very difficult to then go on and use this for the basis of research or treat them appropriately. Another one is it could be demyelination it could be small vessel disease.

I: So how often in your experience do you send patients for additional tests and do you feel the costs are increased due to IF?

P: I think in my practice what happens is that there is rescanning but, that is usually to do with technical difficulties with a scan or, they come back saying we should have given contrast to clarify something or the patient moved and we need to rescan. So rescanning happens for that reason in terms of vascular imaging. I’m happy to factor the images in with all the other things that are going on as a vascular risk assessment. I’m usually less certain that it’s a disease what you are seeing is clinically irrelevant more than the clinical report suggests, so usually I end up saying there is a bit of advancement of small vessel disease for their age but, it doesn’t seem to be that bad and stop smoking and leave it at that. So that doesn’t invoke more imaging but it does lengthen the consultation considerably. With the aneurysm I send them on to other aneurysm specialists and ask them to see them in their clinics and they discuss the risks and treatment options, they will write back and because of admin delays at both ends it can be weeks or months before I hear back and, he usually says the aneurysm is very small but the risk is very large don’t do anything. A systematic review of rupture rates in asymptomatic aneurysm suggesting rupture rates after you have had the asymptomatic aneurysm coiled is pretty much the same as if you had no intervention at all. So you haven’t done anything to improve rupture rate and you have the risks of the procedure. I actually believe the aneurysm question is becoming easier unless you have a very clear reason for treating the aneurysm usually dependant on size you shouldn’t really treat it. And that makes it easier for the patient in a way because they can sense indecision.

P: If they sense that do they insist upon further treatment or imaging

I: Yer yer yer so they can insist on CT angio I don’t mind that if they come up with a plain CT that suggests an aneurysm I would follow it up or if it was apparent on MRI I usually would have ordered a CTA to better define the aneurysm size. Unless I got really good dimensions from the MRI, I think that is important in risk stratification more than a centremeter and I would suggest they got something done about it. There is a bit of reinvestigation that would come from that so they wouldn’t have to talk me into it very much, there is then the issue now, if they have got it do they want to treat it. That is a conversation interventional radiologists have rather than myself. I have had people who are very insistent on having it treated and of course it is up to them. It is their shout but that decision doesn’t happen in a vacuum. It happens in a context of them trying to read between the lines of what the doctor would do and make decisions based upon what they think the Doctor would do or whether they are not being offered the treatment because of the cuts or whatever. I think there is a bit of what the patient wants involved in there but that is not just determinant upon the patient, if the Doctor is confident, robust and persuasive he can explain the risks and reach agreement, you will have fewer people going forward for treatment than if a Doctor presents it as this is slightly worrying or on the other hand the treatment might kill you I don’t know what to do which I don’t think is very good physicianing. But some patients do, I do know of a scenario where a patient presented with epilepsy caused by an xxxx and was offered randomisation of a trial didn’t take it and elected instead for radiosurgery for xxxx xxxx and has ended up with radio-necrosis around about xxx xxxx site and half xxxx head has swollen up and an arm not working and xxxx epilepsy worse than if xxxx had just left it alone and had just done nothing xxxx would have been fine. Sometimes people do get anxious and then it’s more difficult but again what if it’s the patient who is not anxious but a daughter or a parent, this person was xx or xx when he had it done, he was quite chilled about the whole thing mum was very very anxious about it and, she talked him into treatment which was the wrong thing to do, so there are these downstream consequences of once you have been through the loop 3 or 4 times you can sense coming and you can’t with the best will in the world, you can’t tell a patient “just do this believe me I’ve seen this happen before just don’t do this don’t go that way, bad things will happen” and some patients of course are fine with that but others, are don’t tell me what I cannot or should do.

I: Do you find the anxious patients take a lot of time?

P: Eh yer, but it’s worth it if you can get them sorted. You can save a lot of trouble and if they carry on being anxious they will carry on being anxious after the procedure. These will be the ones that have an aneurysm coiled and it will be all right but, there will be a little residual neck and the interventionist will bring them back in 6 months’ time then there will still be a residual neck but, she will come back to me year after year asking if she must have another MRI scan or CTA. Do I need this? So the answer to your question is almost always no, but performing a coiling will not turn an anxious patient into a non-anxious patient it just gives them something to fixate on and worry about

I: Do you hold any opinions and views regarding the management of patients with IFs?

P: I think what is important is to have for them a clear understanding yourself of the risks, whatever this finding is, that is. What I was meaning about white matter disease and how much white matter disease is present and what it means. So a clear understanding of the risk and an ability to communicate those risks in a way that is not sensationalist to the patient and may help them reach a decision on what they want to do or what they want you to do which is unencumbered by their fears and anxiety about their health. That’s the trick being able to present it in a calm and collected way that allows them to set their worry to one side to look at the risks and benefits and work out an approach that work for them. Of course what is right for different people varies and what you don’t want to do is for the anxious ones to be driven into doing everything that’s not good not good.

I: Have you experienced a benefit from an IF?

P: Personally no but…Sure sure if you can imagine a situation where you scan someone from alliance medical at the age of 80 and you find they have a big frontal glioma and they have no symptoms you can say well you may have no symptoms but you have 8 weeks put your affairs in order and they go off to Australia to visit family so you could see that I haven’t come across that in 20 years of practice so I would say that is a rare event. Out of all the events that one could potentially find for how many of them what that knowledge actually is could allow the patient to improve their lives improve their health and Quality of Life which they otherwise wouldn’t have time to do not a lot I don’t feel it happens very often.

I: Do you ever have patients from private companies referred with IF?

P: Sometimes yes, and they are mostly carotid dopplers. There was a place a while ago advertising these and then people came along with 50% stenosis of their carotid, but should 50% asymptomatic carotid disease be operated on again the answer is usually no because of the risks of the procedure. But no, thankfully I don’t get much of those patients referred to me thankfully.

I: Can you see a problem with IF in the future or is it a problem now?

P: Yer well that is interesting. We had a patient xxxxxxx their 40’s presenting with funny events probably migraine and xxxx had been scanned on a 3 Tesla scanner and xxxx had got to my eye a bit more vascular disease and a bit better defined Virchow Robin spaces than you would normally expect to see in a 40 year old person but, then looking at it there were a lot of other things I could see with better clarity and, I’m not sure if it was because it was on a 3T machine that I was seeing the same old vascular disease because xxxx was scanned on a more detailed machine and I suspect it is highly likely that as 3T machines become more common in clinical practice, will we will get more concerned about small areas of cortical dysphasia and slight Cerebellar atrophy with people sent here along at neurology to see what is going on. Again there was a case of a patient with learning disability and epilepsy, the GP had sent them along to CT as if it is going to tell us now about the epilepsy they have had since they were 13 that had been unchanged for the last 5 yrs I mean why why ? What possible use is the CT scan gonna be? They find a cyst or something they then get sent to the neurologist for investigation for what an arachnoid cyst which they have had for a long time or arrested hydrocephalus so as people do more scans especially on patients who have not been previously been scanned we will pick up more of these then they come to us, then we have to spend a lot of time saying well its normal for you it looks like it’s been there for ever its part of you. Like people have one arm slightly longer than the other it’s just how you are put together don’t worry about it, and that all right but at a time where referrals to neurology are increasing year on by 6% for the last 10-15 yrs then that just adds fuel to that and you don’t have to have very many then to add another couple of % to your yearly total. Most of them are pretty straight forward I would be helped if there was a bit less risk transfer by the radiologists and it would be helped also if we were better a having some kind of pre-referral consultation. So taking that example of a patient with epilepsy went to A&E because he banged his head during a fit and then to check he didn’t have an extradural they found a cephalic cyst or something not a completely uncommon story. First thing that could happen is the request form says longstanding epilepsy, recent fall and the scan looks like a cephalic cyst. The report could say in the context of the long standing epilepsy this is unlikely to be of clinical significance and neurology referral is not required unless there are particular issues which you believe warrant their attention. So that would get rid of most of the referrals and then, if we have a pre-referral consultation which we are trying to do. The idea is you see at the moment what happens I get a report from the GP, they say it their report says correlation required consider referral to neurology so I get this very apologetic referral form this GP which says I’ve been looking after this man for 30 yrs and he is the same as he ever was but I have this CT report that says he requires referral to a neurologist what shall I do? And under those circumstances what we could do is we can bang them back an email saying I’ve looked at the scan there is a cephalic cyst there it looks long standing if his clinical situation hasn’t changed no further action is required. That takes 5 mins not 30mins so that raises the question I guess about where a particular service for IF could be warranted that is to say could there be an IF hotline a GP phones up and says I’ve got patient x here is their CHI number is it important? With the radiologist and possibly a neurologist looking at images we can discuss make a decision. You could do that at a Scottish level or Lothian level. It needs a sensible radiologist who is not going to overcall things and a clinician who is going to accept a bit of risk. The idea that you can take all risk that comes in and, transfer it to someone else well huh. Not good not good.

I: Have you anything else you wish to add surrounding the topic of IFs?

P: There is the bit about reporting taking responsibility for reporting I think there’s a bit about access to investigations so we are about to embark on a situation in xxxxxx board where CTA for screening can only be requested by a consultant neurologist so someone comes out of here and they are told that they have an aneurysm so now everyone in the family has to get screened for aneurysm, not an uncommon scenario. So rather than the GP writing to the x-ray department or Joe Bloggs at the hospital for a CTA please, they need to get referred to us. We then have a discussion with them about SAH, aneurysm, risk of treatments and rupture and then they go and have their investigation and that either results in a very short conversation with no aneurysm found and we would already have talked about aneurysm at that stage or if they do have an aneurysm at least then, we have primed the patient as to what to expect and how we take things from there.

I: Do you in your clinical practice explain the risks of finding an IF for clinical patients?

P: Yes, what you do before a test is to assess the impact of an IF. This is something I believe we can do more of. What you should do before any test is to explain the possible consequences of that test and any potential adverse consequences of those tests. So we should be having these conversations with these patients anyway so there should be channelling of requests through specialists so they could do some of that preparatory work for them is a good thing to do. I think for CTA as we discussed it is a good thing to do.

I: Have you experienced any limitation to scanner access?

P: Not here in Lothian but sometimes in other health boards yes. But really, there is no limitation for access to imaging if you can make the appropriate case. For a lot of the tests we would like to request the reason we are told for not doing them is there is not a strong enough clinical indication. When the supply of the tests is not limiting all of a sudden it’s easier to get the test so CTA used to really difficult to get on clinical grounds but now CTA are ten a penny, those clinical grounds have changed, and so has the threshold for which a radiologist will accept a request. I do see lots of practice of limiting scanning due to radiation dose. So IF do affect my work load a bit but not hugely, my take on IF would be that the quasi IF on the elderly population is more evident because they cause more trouble and that’s where clarity of reporting may help. But, Yer technology is finding more together with more and more scanners means more imaging finding more IF.

I: With the range of methods for treatment for pathologies do you ever find patient management an issue?

P: I think the person who is relaying the risks should be someone who is completely up to date, a specialist in the field even so, it can be difficult to always be up to date with what the risks are for varying treatments. I do a scan and I find a low grade glioma in the temporal lobe well I can tell them it’s going to cause them trouble at some time but when well we don’t really know that, because the natural history of incidental discovered gliomas is not great, we’ve lots of information on gliomas which cause epilepsy but not those we just find so we know its gonnae cause some trouble at some point but don’t know when that’s all we can say and, is the extent of my knowledge. It would be nice to be more up to speed with the risks and the potential for what could happen. The patient says what if I’m free in 5yrs does that mean nothing is going to happen, no it doesn’t mean it’s not going to happen it just hasn’t happened yet so I good sound working knowledge of the risks would be helpful and it is my responsibility that I have that but, occasionally you will come across things that you are unsure about.

Participant 3

I: IFs found within the brain are currently holding a lot of academic press at the moment, with patients being referred from clinical, research and private practice. I was wondering what your views were upon the topic.

P: Well I suppose broadly speaking researchers should be made to be aware that and especially non clinical researchers need to be made aware that if they are going to do MRI scans that this is a clinical procedure and whether they are doing it for a patient or a normal volunteer they have to have a system in place to deal with anything that may show up on the scans and not all scientists appreciate that and certainly not all clinicians appreciate that. So that I think is a key point is the need to raise awareness that IF will be found and for each one that is found you need to have an appropriate method of handling it. Which entails you’re having a scan we may find something on it, this is what we are going to do about it if we find something is that ok with you because, I believe it is informed consent and we must make patients aware that we might find things they wished they didn’t know about.

I: How do feel about private patients who opt to pay for scan not understanding the risks?

P: OK privately well that is a separate issue on its own in a sensed from scanning for research purposes because, if somebody submits themselves voluntarily for a health scan or scan of any description brain heart or the carotid arteries in a sense they have asked for it, they have given complicit consent and what the person who is paying for. The health screening company should explain what the implications of submitting yourself for health screening are. So the question you are asking is slightly different but the obligation is similar.

I: Could you describe how IF have impacted upon your workload.

P: Um I think from …as I say I have only had to deal with one that has arisen from a research scan. In routine clinical practice which is the 3^rd^ area we have not covered yet a lot of doctors request scans or are put under pressure to request scans by patients, or by GPs, or other colleagues, it puts us in a difficult position because they have then got to explain to the patient what we are going to do if it has nothing to do with their present complaint. But, is nonetheless an inescapable finding that they have to be informed of so if you like somebody comes along with a headache you say from the headache point of view we haven’t found anything but you have these IF and, we don’t really know what they mean but you at least have to know about them because if you go and have a scan somewhere else the same things will crop up and then at least if you know about it and they are still there when you are rescanned you know that avoids a certain amount of upset and whohaaa. And, I suppose there is a need for awareness amongst the general public that before you submit yourself for health screening or before you pressurise your doctor into requesting a brain scan, just pause to think you may be opening a can of worms you know there are 1 or 2 cases where people have their mental and physical health has been damaged by not fully appreciating that.

I: In your experience have you encountered any benefits from uncovering an IF.

P: Well um as it happens not through my own clinical practice but, a colleague encountered one case where someone participated in a research study found to have a cerebral aneurysm which was treated and one could argue that wasn’t necessary but she then developed some new symptoms which she was quite unequivocal and would not have done anything about had she not known she’d got an aneurysm and went on to have it again successfully treated. That’s about the only one I know off where there has been unequivocal benefit.

I: So in this case do you feel the patient was quite aware what was going on with their own health?

P: In this particular case that particular abnormality a brain aneurysm which was treatable when it later caused additional symptoms could then be treated. But an awful lot of IF are changes in the white matter, small meningioma or small vascular lesion and in general because you don’t know what to do with them, because you really don’t know if they are going to influence health in the long term it is very difficult to give clear advice. So if you compare patients who have say ECG, so if you take ECG’s and do then in elderly people you will finding some people who have had heart attacks who did not know they had heart attacks and there is pretty reasonable evidence that if you do an ECG in an elderly patient you may find evidence of a pious silent infarct you may put them on treatment that may benefit them in the long run. However if you see people who have had a brain scan which shows you’ve got something that looks ischaemic it’s much harder to say if they should or should not go on treatment so the evidence base for things that might not benefit the patient is not the same for the things you might see on a brain scan as it might be for other types of IF that come up with other types of investigation. Of course any investigation can come up with an IF but brain scans are particularly emotive I guess

I: Are you ever put under pressure from a patient to order a scan?

P: Oh yes definitely lots of the time people are…there is a change in society people expect a lot from scans from any sort but particularly brain scans that it will provide an answer to symptoms they have got answer the question and that’s certainly not the case and people also say “ I will be reassured if I have a scan and I know its normal”, the truth of the matter is though that often having a scan and knowing its normal doesn’t make the symptoms go away that led to the person having the scan in the first place I famously had a very fit person who was obsessed there was something wrong with their brain, the person suffered a number of obsessional traits and I said your scan is going to be normal, you have a lot of symptoms that are not indicative of a disease of the nervous system, but the patient insisted they were convinced they did have a disease of the CNS and I said, do you believe having a scan will get rid of your symptoms, he couldn’t answer that question and I said well will you agree that if your scan is normal you will then agree to have some additional psychological help because your symptoms will continue and sure enough they did but he had previously agreed that he needed psychological help for his symptoms and that’s a slightly extreme example but there are an awful lot of people particularly with headaches come along and want a brain scan and are really surprised when you tell them they haven’t got a tumour that their headaches don’t go away. So expectations in society are that A: you can make the doctor do what you want them to do and B: the doctor is your servant no-longer the guide, if you want a scan you can have a scan so that’s one pressure and an increasing difficult pressure to resist couple that with the expectation that the scan will answer the question and you know. Although people having brain scans for health screening thing is not that common. Certainly people have carotid scans from health screening which is crackers, there are companies making money out of gullible people who believe their health can be improved by having a carotid scan.

I: Must you do extra blood tests or anything else?

P: Yes I think as I said undoubtedly if you find something incidental you will need to see the patient again and that is a cost you may need to see them more than once you may need to do additional tests that so yes there will be costs in terms of additional follow up additional investigations and sort of if you like hidden costs the in the any generated anxiety that have been caused in the patient will of course make then possibly seek more medical attention than they otherwise would previously have done so.

I: In what way does the anxious patient affect management?

P: There is prior evidence on brain scans and this much more he case if the patient is a normal volunteer for a brain scan rather than a clinical patient. Back in the 1960’s and 70’s people were offered screening for high blood pressure in their place of work so that they would attend. Factories said ok if you work here we will offer you screening for high blood pressure and what they found was if people didn’t know they had high blood pressure they had only minimal absenteeism whereas the minute they were told they had high blood pressure they thought they were ill and their absenteeism and sickness rates went up merely by being labelled as having high blood pressure and wouldn’t be surprised if there wasn’t as similar sort of effect by somebody suddenly finding they have something incidental on their brain scan it may have costs in that they take more time of work. So their own costs go up and their income goes down because they are taking time of work these are hidden costs I think as well to IF.

I: Could IF affect patients with their life insurances?

P: Yes that is another issue a another cost element umm again it depends upon how it is handled for example if you have a MRI scan that shows a few bits of white matter changes and somebody inappropriately says “ohh you might have MS” that’s clearly bad practice whereas there will be rare occasions when you will definitely identify something with MS who have previously been asymptomatic or was unaware that they had they might have MS. So yer life insurance could be an issue for some.

I: Do you feel the issue of IF will increase in the future or indeed is there a problem?

P: Um I think it’s not going to go away and with higher field strength MRI and increase image resolution it may get worse because obviously you will have higher resolution to detect small things and the other thing to some extent it may get better as we begin to understand what happens to people who have IF discovered on a 1.5 or 3T scanner but it might be that if you start scanning people with 3T you discover a different sort of IF and its gonna take a few yrs to sort out what we are gonna do with them, because one of the things that is quite helpful is that you follow up patients with IF and see what happens to them and we haven’t accumulated that experience yet, so I don’t believe it is going to go away its likely to become and it’s not going to be any less of an issue there is scope for it to become greater but it is hard to predict. Possibly more MR scans will replace CT scans. It is quite possible because of the radiation issue associated with CT, so there will be a greater likelihood of people having an MRI scan in the first instance increasing IF.

I: Do you in your clinical practice explain the risks of finding an IF for all patients, there have been discussions in academic papers about tackling patients before they have their scans what are your thoughts?

P: Yes definitely. I think that anybody that is having a scan needs to be given an information leaflet and encouraged to read it both for CT but for a greater extent MR. Having a MRI scan for some is a slightly unpleasant experience but I think understanding the consequences what will happen if there is an IF raising the possibility so you perhaps need two sorts of information leaflet one where which specially deals with IF where the Doctor can say look in your case I believe your scan is going to be normal but I am not keen to do this scan because you may uncover an IF, I’d like you to read this leaflet and let me know if you still want the scan. So, if you like that could be a fairly graphic potential aspect of consequences of an IF and then you’d need a slightly more general MR info leaflet to deal with what it’s like to have a MRI scan. But, if you are investigating somebody for a specific problem you turn up something else well I think that’s slightly different from if you are not expecting anything and you turn up something which in your opinion is nothing to do with their symptoms and is not significant but then you have an obligation to tell the patient. I think that is theme which is an obligation to be completely open with the patient you might well get into trouble if you identify an IF and don’t tell the patient about it. You know a paternalistic approach its only x the patient doesn’t have to know. I’m sure that happens doctors make a judgement which may have long term consequences they hadn’t quite anticipated ..Why didn’t you tell me all those years ago that my scan showed something funny. You know.. I think people and doctors need to think very carefully about making sure they are open and honest about what on the scan report with the patient.

I: In your experience have you found any issues with managing patients found with IFs?

P: Do you mean what do what you do with aneurysms, white matter lesions and such?

I: Yes

P: I think by and large for most of the common IF: white matter lesions, vascular lesions, aneurysm, small tumours there isn’t a solid base evidence treatment to do with any of them. If your aneurysm gets above a certain size or if you have an AVM again there is no good evidence with how to deal with it I think even for the specific IF there isn’t any good evidence based practice for any of them.

I: Do you ever find that difficult for you with these patients in front of you wanting advice for their IF

P: Yes yes it is difficult because you have to admit that you don’t know and admitting uncertainty to patients is not something that I mean I don’t have a big problem in saying you have an unruptured aneurysm its v small we don’t know what to do with it on the balance of probabilities is best left alone but there isn’t high quality evidence to tell us what to do with it in a sense I don’t have a problem with that because I am familiar with evidence based medicine and I don’t mind if there isn’t evidence just talking to the patient with what information there is but it is always an awkward conversation both for the patient and the Doctor both like to know with clear evidence what to do but as I said there isn’t any good evidence for any of them.

I: Have you anything else you wish to add surrounding the topic of IFs?

P: It is a difficult area to educate the public in because you know there are more important areas of if you like public education. But it is something certainly that the health service and people who provide scanning services need to highlight to their clients and there probably scope for the government to regulate the health screening industry a little more carefully than it currently does, although I know it’s difficult to legislate for anyway you know it is an interesting area it is a problem it is not going to go away. If we can give information to patients it would be really helpful.

Participant 4

I: IFs found within the brain are currently holding a lot of academic press at the moment, with patients being referred from clinical, research and private practice.I was wondering what your views were upon the topic.

P: Yes lots. The best paper around is the systematic review published in the BMJ so yes I am familiar with this literature. I had looked into one of the Dutch studies for a journal club which looked into IF and had done a couple of talks on this subject. So it is something I have been interested in for a little while. I am not completely up to date with literature but reasonably aware and I think any practicing neurologist has to be aware of the problem, we can’t not be aware of the problem. We are all aware of it but our approaches to it vary a great deal. I think we all realise it is an issue.

I: Could you describe how IF have impacted upon your workload

P: I guess what you mean is in the generating of referrals from IF..yes..the problem is none of us have systematically audited it my perception is that in terms of numbers it is probably not a huge issue in that half the clinic is not full of these patients. so numbers wise it is not that much of a problem but I think that in terms of individual patients it is a problem there is a great deal of angst alarm and concern. It does of cause generate extra work particularly radiologically because some people may arrange follow up scans. I think at the worst end of the spectrum it can lead to inappropriate diagnosis and even inappropriate intervention. For example a headache patient with a Chairi, a fairly common scenario can occasionally end up with foramen magnum decompression. Now that wouldn’t happen here but I have frequently seen patients with surgical interventions for what I think are incidental finding. So yes I do think there is a problem but I think someone would have to systemically measure it to tell you what the size of the problem is rather than relying upon anecdotal things like I’ve seen a few things. My perception is in raw numbers of things it is not a huge problem although I think it has the potential to become a problem as more patients get imaged as they surely will do and as imaging technology improves and picks up more and more subtle features

I: Have you had experiences of pseudo disease and inappropriate diagnosis

P: Yep so scan first and then symptoms later, very definitely not infrequently these IF are the ones that there was not much wrong to start with if you see what I mean and of course, they are as worried as well and some people do a scan just to reassure them and you don’t reassure infact, you do quite the opposite then you cause great fall out and I can think immediately of 2 or 3 examples where there was enormous fall out. And yes, they did develop all sorts of symptoms do you think this tingling l’ve had in my right finger is related to my IF and so worth and so they do undoubtedly induce anxiety and then generate physical symptoms causing more angst

I: In your experience would you say patients have insisted upon an MRI.

P: Oh yes, insist is maybe too strong a word but even some straight forward patients you always have the conversation where you say this is what we see you don’t need further tests and they say but I want one. For a NHS patient I would explain this is not really appropriate but privately phuuuu the attitude is “well I’ve paid my premiums I am entitled to my scan”. For some time now when PP have asked for a MRI scan I will warn them of IF and very few patients understand what you mean that by finding something incidental may ironically cause you more angst and not reassurance which is what they were looking for in the first place. I briefly consider a CT scan but then you are exposing them to radiation while as far as we know MRI is completely safe and secondly it may sound stupid but patients know that MRI is better than CT and a lot of them see through that so it’s a nice idea to do a CT and then we will hopefully not see any white matter change in the elderly but it doesn’t work that way.

I: In your experience how often do you feel the necessity to request further imaging or intervention?

P: Where IF have cropped up?

I: Yes

P: It depends, usually the commonest IF are Chairi, and arachnoid cysts which by and large you can say do not require tests not even imaging let alone anything else. More difficultly white matter changes in the elderly again you can normally reassure them that the ones where you end up doing further investigations are either when the nature of the IF abnormality isn’t clear then you need further imaging so that one aspect where you need to rescan them in 6 or 12 months ditto with meningioma’s which are uncommon. With meningioma you are bound to image them 2 or 3 more times almost bound to they almost never change but you must. The other difficult one is young patients with white matter changes with the question odd demyelination and we get a lot of those from ENT. They go to ENT with their dizziness do an MR find some non-specific white matter change which you don’t feel is relevant but do you need to do a lumbar puncture do I need further imaging or repeat imaging a few months on so yes there can be cost implications because of further investigations.

I: Do you hold any opinions regarding the management of patients with IF?

P: Well I think by and large it’s fairly straight forward. The older patients may have got some hypertension some-one has requested a scan for nebulous reasons and they have white matter changes. There is literature now purporting that this now predisposes you to stroke, dementia, premature death and so forth. Should those patients be treated in the same way as someone who has presented with a TIA, should they be started on an antiplatelet drug a lot of people do I have to say I don’t because there is no evidence to support it and there is no evidence not to support it so there is a list of studies that haven’t been done. Of course we treat hypertension when it arises as well but the evidence for using aspirin for intervention suggests it does more harm than good but that is for most people but not the population we have just talked about. There is uncertainty so there is that dilemma. Aneurysm they are not a common IF but we do come across them the bottom line is there is no evidence base for treating unruptured aneurysms and we know fully well that sometimes things go wrong and we can wreck them which is a disaster. The fear is the trial is never going to get done. That is a dilemma with the potential for harm. There are areas where it is clear arachnoid cysts, but there are areas Chairi, white matter changes, aneurysm where you are much less certain meningiomas we tend to follow them up and maintain surveillance on them, they have cost implications but not treatment implications. Low grade gliomas which are uncommon IF but they do occur but by and large most people will agree to keep those under surveillance and then the kind of non-specific ones something not quite right on the scan they don’t know what it is where again you are going to have to follow it up with surveillance so it’s not a treatment dilemma but a management dilemma they are plenty of areas of uncertainty about what on earth you do once these things arise because of course if you knew what to do you wouldn’t be so worried about it.

I: Have you ever come across any be benefits from an IF being uncovered.

P: Of the top of my head….I don’t believe so. .. No. I can’t think of any circumstance where I was pleased we found that no quite the opposite.

I: How much of your time do you feel patients with IF take up?

P: Some patients get very worried often because well there are two scenarios doctor asks for the test, I get the results I manage the patient, then if we return to the ENT scenario white matter change and gets told by the ENT surgeon sometimes by a letter or by their GP oh there is something wrong with your brain scan it might be MS or that is what the patient hears. Then they can be waiting 4 months to see us in that 4 months they can crescendo into an acute anxiety state by the time they come to see the neurologist they have convinced themselves they have MS and you them have to try and dismantle that and it is not going to take 10mins and in fact the irony is the patients always seem to take the word of doctor not specialised in neurology which can be very frustrating. It depends upon how the information is fed to the patient …some of the stories are awful I just got this letter or my GP just rang me up that may not be true you may find the patient had a sensible conversation with their GP it’s just that how the patient perceived it. And of course the GP is not an expert in this arena you can’t expect a GP to know all about MS, the chapter and verse about the significance of a little white matter changes on an MRI scan when they didn’t even organise it. So yes I think IF patients take longer consultations.

I: Do you find you get any more referrals from any one source?

P: I have to say ENT has cropped up on more than one occasion because they do seem to MR scan on most patients with dizziness it seems anyway. No I don’t think so most specialities. I think this is the way modern medicine has gone if you over investigate people you will uncover more the VOMIT theory (Victim Of Medical Imaging Technology)

I: Can you foresee a problem regarding IF in the future?

P: Yes yes unquestionably and it is for reasons that we have discussed before firstly because more patients are being sent to specialists secondly because there is greater access to radiology. I arrived in 1992 and there was an 18month waiting list to get an MRI scan and only a consultant can order it now GP can organise an MR or an FY1 sitting in an emergency room can request one yes, there will be more investigations the images are improving year by year and therefore you are picking up more and more subtle findings it unquestionably going to increase.

I: How do you feel you will manage this demand?

P: I guess we need to find out some answers to some of these questions so let’s worry about the big ones like white matter changes which is the commonest so it would help what to do if these are found in the healthy and what if any treatments should be instituted now that going to require some fairly large scale studies. So if we could find out answers to these questions we might be in a better position to answer patients’ queries about what does this mean, what shall I do. I tell you for instance insurance companies will load the premiums. I found this out a few years ago with a patient in their mid-forties who had a nasty headache which we both agreed to scan and he had some relatively subtle white matter changes a bit more than normal than he should have for his age but nothing in particular and he then forwarded me a letter from his insurance companies which had doubled his insurance premiums from the year before on the basis of that finding. And it coincided with a letter in the BMJ which suggested that white matter changes were connected with dementia early death so the insurance companies have locked into it. That’s another way how that can affect the individual its going become a more frequent problem, we are living longer we are more inclined to investigate older patients than we were before you’re more likely to get referred to specialists that we were before so inevitably we are going to start tripping over more

I: Are you happy with the format of imaging reporting in for example white matter?

P: I was very happy to see a change in reporting from white matter disease to changes as soon as a patient sees the word disease if freaks them out because we do not know yet if it is a disease. Reports made by non neuroradiologists may have more margin for differing interpretation.

I: Do you believe patients with IF could be managed differently?

P: Well the question is how, because they can be managed in all sorts of different ways. well I’ll answer it for my own management of patients if I had all the answers for the questions we discussed before I suppose I could but as I don’t I can only manage them in the best way I can which I believe I do so I don’t wack everybody on aspirin for instance and some people do it would be helpful to know if they are right or if I’m right.

I: Have you anything else you wish to add surrounding the topic of IFs?

P: You do feel we create more problems than we could solve and that whole “VOMIT” idea –“Victim Of Modern Imaging Technology” thing it’s the price you pay I think and you have to agree modern imaging has given us a lot more positive and negative and you don’t get something for nothing and we just have to find a way to deal with it you just get sanguine about it. There are stories from the US about some people suing because of IF and they weren’t warned about it in the first instance. Some patients mainly PP don’t get the warning about scanning without good reason they simply don’t they look blank and say but that what I want if you find something I can do something about it. But I will have to tell you if I find something even if it is totally irrelevant the days where I didn’t worry you of a finding which was prevalent in the past do not exist now so you have to tell patients which may cause them more worry I do think we have to be increasing aware of IF and that we have to warn patients and we have data on figures there is a 1 in 20 chance we will find something hard data now depending on your age. But if we do enough scans we will uncover more IF. We do trip over meningioma’s once or twice a year they are frequent enough to notice it’s not once in a blue moon. Naw you just get sanguine about it.

Participant 5

I: IFs found within the brain are currently holding a lot of academic press at the moment, with patients being referred from clinical, research and private practice. I was wondering what your views were upon the topic.

P: So my thoughts are as a clinician that to be honest in terms of people being referred to me with IF that has happened rarely. In terms of me investigating my patients and finding an IF on the scan that has happened occasionally. I am a relatively junior consultant neurologist and in my personal experience so far IF haven’t caused to many problems, although I can see how it could in terms of colleagues this has led to difficult discussions. So IF come about in two ways either when you are doing a justifiable test and you find a finding that is unexpected you might call those unavoidable IF because you were going to do the test anyway and then you’ve got the avoidable IF when someone comes requesting a test which happens quite frequently.

I: Do you in your clinical practice explain the risks of finding an IF for clinical patients?

P: I do not feel the need to discuss IF with patients presenting with sound justification for scanning, but yes those who insist I will detail the risks of IFs.

I: Do you ever feel under pressure from patients to give them a scan?

P: Oh yes that happens very frequently, they come in with headache. They will have gone to their GP and requested a scan because they think an appropriate examination for a headache is a scan of the brain, it is very difficult for the GP to deal with that. Then they are referred to my clinic and we have a discussion which is either satisfactory to me and that is I seem to have reassured the patient that they don’t need a scan. Whether or not they are reassured I am not sure ..or unsatisfactory where I don’t reassure them to avoid a blazing row we arrange a scan.

I: That appears to be a lot of pressure

P: Oh yes, you are always under pressure aren’t you.

I: Have you ever experienced any benefits of uncovering an IF

P: Hmm. Its pretty difficult to think about them being beneficial within the brain um so if we think about the common IF which are white matter changes. I suppose the benefit there could be they had changes in their brains related to their life style they might be able to change it to but I don’t think I‘ve ever experienced anyone doing that. In terms of brain tumours my view is that any uncovering of a brain tumour is harmful rather than beneficial because treatments as a whole are unsatisfactory. I mean if there were curative treatments for brain treatments then that might be worthwhile. For aneurysms’ well…hmm that question is really about screening isn’t it? If the question is asked are there any benefits to uncovering an IF that’s a screening question well they don’t. I mean in mammography for example screening for breast carcinomas they weren’t causing any symptoms there is a lot of controversy whether that is beneficial or not they are if you like IF but deliberately sort and within the brain there aren’t meant to consider really and I can’t think of anything of the rare thing that could possible turn out to be found that could be cured. So, no I can’t think of any.

I: Have you any experience of any deficits caused from uncovering an IF.

P: I suppose my experience is limited but I could give you examples where people talk about them as neurologists and have found an IF and so have an AVM or aneurysm and clearly if you have been told if you have one of those things and you were worried at the beginning then clearly you are going to carry on being worried. If you are the kind of person who has been pushing for an investigation then you are likely to push for treatment and I can certainly give examples of people who have been harmed by those treatments but as yet and I’m sure it will but it hasn’t happened to me where a patient I have directly looked after as one of mine has encountered this. Other harms well, it’s difficult to gauge this physiological harm as I don’t follow people up in the longer term.

I: In your experience how often do you feel the necessity to request further imaging or intervention?

P: Rarely, but as I said I don’t see many patients with IFs.

I: How much of your time do you feel patients with IF take up?

P: Depends really as you know some people are anxious types and will take longer to consult and some people are quite pragmatic about things it really depends upon what kind of person they are to a certain extent. For some people the IF creates great anxiety quite reasonably so and others are not bothered by it.

I: Do you foresee IF being a problem in the future for yourself and causing any cost impacts for your practice

P: Yes there is no doubt that IF are frequent and the impact they have is chance rather than anything else then I suppose you come to the thought that one persons IF is another’s cause. I mean if you went to speak to certain doctors they would call it the cause of their symptoms I bet there are plenty of neurologists on Harley St who would explain that as the cause for their symptoms.

I: In your experience have you found any issues with managing patients found with IFs.

P: I suppose there are two questions there. How do we manage particular IF for example aneurysm and I think that recruiting patients to those kind of trials is very difficult so whilst we don’t have any, it is difficult to imagine how we would get people into those kind of studies given that there are two studies on at the moment. One is the STOT team trial on asymptomatic aneurysms I think that has stopped and a trial investigating AVM’s and both of those struggle for volunteers… so how you would get that information I don’t know. The second thing is about long term prognosis simply an observational study of IF that might be interesting but difficult again to get numbers and the third thing is how would you manage the anxiety caused by tests, sometimes of course it might be justified we simply don’t know.

I: Have you anything else you wish to add surrounding the topic of IFs?

P: Only, the problem will grow as we have more scanners running so, what this space.

Participant 6

I: Incidental findings found within the brain are currently holding a lot of academic press at the moment, with patients being referred from clinical, research and private practice. I was wondering what your views were upon the topic

P: Just within the brain or including the spine too?

I: Just the brain please

P: OK, that’s a shame there are a lot within the spine too. Firstly, I don’t do private practice but my general views are that they are a big problem for us clinical neurologists. Both in terms of creating referrals to the neurology service that otherwise wouldn’t happen and, also in investigating patients particularly those that I see that have functional symptoms and don’t have any neurological disease. There is always a risk you are going to find an IF and those patients in particular will tend to latch on to this IF as an explanation for their symptoms. And it massively creates a huge obstacle for treatment for these patients and takes a lot of effort to get the patient around to our way of thinking, that they do have something that is potentially reversible as an abnormality on the scan and if its nature as an IF is bit unclear then they latch onto it and believe that they are damaged. Therefore it’s much harder to improve so my own practice now for IF is v much to anticipate them. So like yesterday I needed to arrange an MRI scan so, I explained by the way there is a 1 in 20 chance we may find an IF of a cyst or some white blobs or something that has probably been there since you were born that is not the cause of your symptoms so I think anticipating them helps when they do crop up. That’s how I try to manage them and they are a problem and I particularly think the better the scans get the more detailed they are the more we are going to see IF.

I: Have you noticed any subtle differences in imaging resulting in further questions due to the advances in imaging technology and higher field strength scanners?

P: No not yet. I had a patient who had enlarged peri-vascular spaces –Virchow robin spaces. Which has traditionally been regarded as an IF- this patient found some research from this department actually that indicated there may be some correlation of developing MS. xxxx really latched on to this and something more than an IF perhaps xxxx is right perhaps these things are more than an IF and we should worry. But trying to keep pace with what these things mean is difficult as well.

I: How much of your time do you feel patients with IF take up?

P: Umm, well my patients do take longer as they have functional symptoms …some have 15 symptoms and have seen 10 consultants already and come with a feeling no-one believes them it’s all in their head so it’s just an extra layer of complexity to add on which if you had a scan which was normal or abnormal then it would save a few minutes..but that not’s the way the world is .. so yes it does adds to the complexity.

I:Have you ever felt pressurised by a patient to request a scan?

P: All the time and I think quite frequently I manage to prevent it and say the department money but of course you don’t see that you only see the ones where I’ve not been able to so for headache particularly its very often clear that the patient does not need a scan. They have migraine which has been going on for 2 yrs and I would say to the patient definitely from my point of view you don’t need a scan I’m confident about the diagnosis and if we do a scan there is a risk we are going to find an IF. But depending on the patient it is pretty clear sometimes that the way they are behaving they are even disappointed they are even having a chat with the neurologist what they thought they were coming for was a brain scan for their headache and that’s what they want. If that seems to be what is standing in the way of recovery then I will do it.

I: Could you describe how IF have impacted upon your workload.

P: They are common enough that we have to think about it how much I have to think about them compared with how much research is done about them there is a big disparity I should say. What does it mean when someone has a dilated Virchow Robin spaces- ummm is it really one blob per decade, there isn’t much literature to tell me do arachnoid cysts present no symptoms, occasionally some people say they do, but no they don’t hugely but it’s a definite significant problem and they certainly present work for new referrals particularly ENT. You have probably heard that before eh? Someone comes in with tinnitus and a slight hearing problem has a brain scan and leave with an IF.

I: Do these patients get consulted about these findings by the ENT doctors?

P: I don’t know what the ENT doctors do, I never seen a letter saying I’ve seen an IF to the patient but they might have done but, my guess is talking to these patients is that they don’t warn them at all about these findings which is bad really, I mean there is what a 10% risk of finding something, that quite a high chance of finding an IF and I believe you should tell patients of this risk.

I: In your experience have you found any issues with managing patients found with IFs.

P: Umm. Well aneurysm are pretty straight forward I just send them to the aneurysm clinic but that’s passing the buck really isn’t it umm white matter lesions are particularly difficult especially the ubo’s (unidentified bright objects), I had a patient the other day I sent them with their scan and said your scan has a few dots here and there which is completely normal .. a normal scan I was very explicit with her saying that it’s a normal scan. They are just ubo’s, I don’t know what people think when they hear that. A very anxious patient will go away thinking I’ve got ubo’s and not hear the last bit. When there is a lot of white matter changes than normal not just a few bits it is difficult. I mean sometimes you see a scan that is very typically an MS scan and then you have to explain to the patient and since the literature on this topic is not great some studies say that 30-40% of these patients who present like this will go on to get MS you have to say I’m sorry but you do have a risk of developing MS. We do have these sorts of conversations with patients who present with one episode of MS ..you don’t have MS yet but you may have a 50-60% chance of developing it in the future and that’s a very difficult thing for people to hear. But if you have never had any relevant symptoms and you are suddenly told this well it’s a bit weird for them. If you just went along to the clinic with reduced hearing and then all this huh often if you just have vertigo vestibular well its nothing to do with then brain so you just have to tell them there is nothing you can do about it clinically you just have to see them again. Then there are the patients with white matter changes that looks like hyper tension problems well let’s check your blood pressure and cholesterol, that’s probably a bit more useful because if you do see an abnormal scan a sign of something that is not quite right. But there is literature about migraine and white matter changes as well making things tricky and depression and white matter changes as well so if I’m seeing someone with functional symptoms and they have migraine and depression and they have a bit of white matter changes I don’t know if that’s meant to be there or what it makes things very difficult. We need to know more about what this all means for individuals it is going to be hard to do that. And other things like cysts and caveromas I will ring up xxxx (another specialist in the field), arachnoid cysts I’d say you have always had that- good line. You would have had that even when you were a baby- it assures people. It’s part of you, it’s the way you are.

I: Have you experienced anyone with meningioma?

P: Oh yes lots of those lots of elderly patients with meningioma which is difficult because this is a brain tumour and everyone gets pretty alarmed they are not usually symptomatic either. The older patients aren’t so bothered about it they say ok you are the doctor if I’m not to worry that’s fine. The younger ones will go away and google it, the internet has its goods and bads.

I: How do you feel IF will present more issues for the future?

P: Yes I do. With technology improving resolution improving and the possibly of field strengths improving even beyond 3T to 7T yes I can see the problem increasing. With more deep venous anomalies showing and variations in normality that people will pick up on and start associating with things like the dilated Virchow-Robin spaces ..I had previously disregarded them as normal simply incidental but now looking at research maybe they are not, maybe I should be worried about this patient yer I can see them as a problem. I mean it’s kind of inevitable isn’t it I mean the only solution would be not to have 7T scanners yer know or just not scan people unless you really can’t help it and just become much more aware of the dangers of scanning. We must do even more studies on healthy people to find out how common these things are.

I: Are you experiencing any cost impacts upon your practice

P: Yes there is in terms of these referrals that come specifically for IF and so that the more that we have open access to CT for the GP I’m sure we will see more of this coming along. It really a problem of the ordering of the scan when they are not able to cope with the consequences of it, like the ENT lot having a scan of the whole brain when they are only interested in the ears, and headache requests where they send the patients of for a CT scan and the result comes back of an arachnoid cyst no what do they do with them, costs are likely to go up.

I: In your experience how often do you feel the necessity to request further imaging or intervention?

P: Oh often, but I do try to limit it, but I bet you don’t see it at your end eh?

I: Do you receive a lot of referral s from the GP’s.

P: Most referrals are from the GP probably about 90% come from that source.

I: Do you believe patients could be managed differently, we have talked about explaining about the possibility of IF before there scans, do you feel there are another ways that could be used

P: Well I think that’s the very most important thing and I’m not sure all my colleagues do that and I don’t always remember I do forget on occasion to mention it. It should be a really standard routine part of any consultation before agreeing to a scan because it is such a common problem. There is quite good research available that if you anticipate certain results with patients it significantly reduces their anxiety about having the tests results and then waiting for the results. I quite often say to patients look I’m going to have to request an MRI scan of your brain here because I want to make sure you do not have any disease as well as functional symptoms I think it is going to be normal I will be very surprised if its not normal but there is a 10% risk of IF that may happen. If you set the scene like that when you come to the next consultation they say that what you said may happen then its normal apart from the IF. So everyone is calm there is no shock with the revelation of say a cyst in my brain

I: Are there any other issues you have that you may wish to share

P: Functional imaging, that’s a problem and is going to be a growing problem so I had a patient recently who has got whole load of issues, pain, hemi paresis and more and her GP had sent her to a neurophysiologist rather than me and unsurprisingly she performed very badly which is what we would expect but the neurophysiologist said whoaw what is going on here and referred her on for fMRI or SPECT there is no such thing as fMRI as a test but there is SPECT and there is tool for finding out things the analysis which I feel is very dodgy for cognitive problems anyway and so there is a whole other growth area of finding abnormalities or anticipating them. Perhaps in 5 or 10 yrs time we will be using fMRI but without an adequate knowledge of what it’s supposed to look like for a whole range of conditions in normal people. I can just see these new technological advances being a huge problem.

I: What do you think of tractography?

P: Oh yes I’ve got abnormal tracts. Psychopaths have abnormal tracts you might get a report back that says your tracts look like that of a psychopath. It’s all about just doing the research really what things mean, it doesn’t mean we shouldn’t look but I feel it’s not been given enough thought.

I: Do you feel IF impact on the patients QoL

P: Yes I think they do the ones that pop into my head are people with aneurysms, the patient is told about them and they sometimes feel that they have a potential bomb in their heads they may explode and kill them that’s definitely likely to impact upon their QoL. If you are of a certain disposition well I mean for some people its fine if you tell me its fine then I’m happy with that its fine. Others people with health anxieties can’t let these things go even if they want to, even if they know they should they can’t.I’m going to get MS I’m going to be disabled and in a wheel chair the scan showed these blobs so yer definitely.

Participant 7

I: IFs found within the brain are currently holding a lot of academic press at the moment, with patients being referred from clinical, research and private practice.I was wondering what your views were upon the topic.

P: I think they are a big problem in other words the issue of them is being more frequent and it’s a problem in clinical practice as well as in academia and I think the problem is not just in their detection but it’s all the things that follow from that so the clinical implications the uncertainty about what is the right thing to do regarding the IF and also the logistical consequences of detecting an IF in which differs from academic and clinical work and the ethical ramifications for patients who perhaps weren’t aware that an IF could be discovered and I think that then has consequences for what really preceded the detection of finding in the first place how they came to have a scan and what they should have been told prior to the scan being done. So as I see it those are the major areas that are problems with IF.

I: Could you describe to me how they have impacted upon your workload

P: I’m particularly interested in vascular malformations of the brain which are quite a common IF and being interested in stroke.. the reason I am interested in vascular malformations of the brain is because they are quite often detected without ever causing a stroke due to a bleed and one of the dilemma is should they be treated to avoid a bleed happening in the future or if the treatment itself causes more harm than good but obviously it’s an even more tangible problem when someone has had a bleed from one of these and then the dilemma is whether they can treated and will that ultimately do more good than harm. So as a result of that clinical address that lead to a research which is of interest to me here in Scotland to do my PhD to better understand the risk of these vascular malformations so then having taken a clinical interest in them and a research interest in them I am now leading the service for people found with AVM’s of the brain and cavernous malformations of the brain leading on these patients clinical care and I’m now xxxxx xxxxx has asked me to be the neurologist who sees patients with IF discovered during research imaging in the NHS the good practice recommendations for research participants is if a significant IF is discovered there should be clinician to whom that patient can be referred so I am that person so but so basically the impact on me is a number of clinical and academic aspects of my life in fact they are probably a very large part of my everyday practice

I: How much of your time do you feel patients with IF take up

P: So I do a AVM clinic once every month, a cavernous malformation clinic once every month and the multidisciplinary meetings that are associated with those clinics and there is a fair amount of administrative work relating to the patients who are seen within the clinic that’s the clinical involvement as far as the academic involvement probably about 50% of my time that’s 50% of my academic time because my research interest is those conditions

I: Could you describe how patients with IF present to you

P: Usually they arrive pretty uninformed or completely uninformed in clinical practice a scan is done because they have clinical symptoms which might be due for example a brain tumour or MS and then something is found incidentally exactly what the original clinician does about that finding does vary for example good practice might be that that clinician brings the patient back to the clinic and says by the way there is no brain tumour or MS but we have found blah this here is some information about it I am going to refer you to a specialist to discuss in more depth what the next step is so that might be a well informed patient but the vast majority of the time the scan is done the result is relayed to the patient by their GP and the clinician relays the patient to me and asks me to see them in clinic so either the patient gets an appointment letter from the clinic and are asked to come and see me not even knowing why they are being asked to see me or they go and see the GP what this is about and their GP says this quite a rare problem I don’t know what it is you had better wait and see the specialist or as I am increasingly doing now so I am not taking hostages to fortune I receive the letter from the clinician and I write to the patient saying Dr Bloggs has referred you to me this has been identified on your scan here is a patient information leaflet about this condition to give you some information and then come and see me so in short a common experience for me is that patients are relatively uniformed and my job by and large is to give them information about their condition and then tackle the situation as to whether or not they should have treatment and it seems to me that this is an inefficient use of my time an resources to be giving the patient information during their half an hour appointment with me to bring them up to speed so that they can then make the difficult decisions about the treatment its more worthwhile for that half an hour on the part of the patient to spend the time discussing their condition looking at the scan which is what I do with them ill show them what it looks like on the scan discussing the significance over and above what they have received in the patient information leaflet and then focus on the nitty gritty of whether they should have treatment or not or what their plan is or any life style implications for them and so on . So by and large patients are uninformed quite often frightened because they don’t know what is going on and nor does there GP quite often they are frightened because they hear there is something abnormal on the brain scan but they don’t really know what it is so that lack of knowledge breeds fear which is also a significant part of what I have to do – allay fear whilst at the same time appraising them of the risks of the condition and laying out the choices they have in front of them but if during your consultation you end up educating them and informing them and explaining and then being left with 5 or 10 minutes on treatment by the time they have received all that information they are not in a state of mind where they can actually cope with making a tricky decision about treatment so they often have to go away a think about it and then see me again or call me back or whatever. I think one consultation when you are going from a point of them having no information to them being fully informed and then making decisions about what they have come to see you for that’s pretty demanding on any individual.

I: How often do you suggest further imaging or intervention?

P: Further imaging is sometimes required for clarity of the IF so when somebody has are research study quite often there might be a bunch of sequences like an fMRI has been done with a standard T_2_ or T_1_ axial that gives you a bit of information to detect something but in comparison to clinical imaging doesn’t give you all the information you need to say what exactly this IF is and how it is put together so in those circumstances we are often asking for further imaging to clarify the IF better in a research context sometimes in a clinical context for example the patient has neurological symptoms and the MRI request is put in to investigate the possibility of MS. MS orientated sequences and parameters are chosen to best visualise this, the scan is done the report is written and sent away and later let’s say there is a cavernous venous malformation well GE imaging is relevant to them but was not done at the time and is useful to determine if there is only one or if there are many so even in the clinical setting if a scan is used to see x and y is found frequently y will require further sequences to identify it that aren’t done because radiologists aren’t always viewing the scan whilst it is being done and the patient is in the scanner so what proportion of the time would I request further imaging 50-75% of the time some kind of that ballpark figure it does depend upon what kind type of abnormality it is

I: Must you do extra blood tests anything else?

P: Not blood tests, the usual requirement is for extra imaging. though for cavernous malformations, although I have already described that case I take blood tests for then because if you have multiple cavernous malformations you might be the carrier of a genetic mutation that has a 50/50 chance of being passed on to your offspring so knowing whether you have one or many cavernous malformations may have genetic implications. Then there is the whole separate discussion about the genetics of caveromas to be had at which point genetic testing may or may not be undertaken by the patient so usually not because cavernous malformations are normally benign disease that normally people don’t do much about but usually its imaging so if you take an incidental aneurysm finding or an incidental AVM discovery then quite often MRI or catheter angiography may be appropriate especially when it comes to putting patient into low medium or high risk groups with respect to their future prognosis of the vascular anatomy of these things and the vascular size, aneurysm location in the circulation of the brain in addition to age and that sort of thing will have a difference with the bleeding risk of an aneurysm whereas an AVM needs to know the pattern of venous drainage whether its deep or superficial or whether there are aneurysms on them or not may influence the perceived future prognosis so that further test that I ask for is often done with them when they are found on CT or MRI in order to give patients further information about their prognosis and then the decision whether or not to treat them follows and if the decision to treat is made then there is a whole host of further investigations and expensive treatment and follow up that follow. The costs for the NHS just wrack up more available MRI in clinical practice easier it is for clinicians to obtain it because it doesn’t involve radioactivity therefore clinicians will request an MRI because it is safer than a CT but you are more likely to pick up an IF so the more available MRI is the more MRI is requested and then there will be more IF discovered

I: Could you describe the costs to the patient.

P: Sure there are implications for the patient sometimes in terms of their driving license especially if they are a type 2 vehicle license holder a long distance lorry driver for example who will lose their type 2 license if they have an aneurysm or AVM till its blocked off there are economic implications for them and if the patient has seen commercial or private practice then rather than the NHS then there are cost implications for that for their insurance policy or if they paid their own way to see someone in the commercial sector yes and time anxiety all sorts of costs studies have been done showing the anxiety the way the results for patients with these incidental findings vascular finding and that sort of anxiety may cause mental health problems which will impact upon their productivity at work

I: Do you ever feel pressurised by patient to request a scan?

P: Yes for people who want a scan because they have a headache to seek the cause my way of handling that is to good communication with the patient take a really good history try and work out what the headache is due to examine them inform them about whether their examination is normal or not if their headache sounds like migraine and their examination is normal then a good communicator can do a lot to reassure somebody that their headache is migraine and migraine is not related to underlying abnormalities the vast majority of the time but there are some people that despite that or if not confronted by a clinician who communicates reassuringly way will still ant a brain scan and at that if they are so utterly insistent on it there are two options if they have come to secondary care having been referred by their GP it’s my decision whether to request a CT or MRI if the patient is seen in the community there is this whole debate about direct access to CT scanning if they go and see their GP and say I am worried I have a brain tumour the easiest thing for a GP to do is to put in a request for a CT rather than refer to neurology for an opinion knowing the patient may not be seen for 3-4 months CT they can get within a few weeks so the handling of that problem depends upon the context that’s the usual primary care response but again really sensible GP will defer the need for imaging to us if there aren’t any red flag signs headache with worrying symptoms but when they come to us any decisions on a scan then a will tell them about the randomised control trial that has been done in the JMP by coward I think where they randomised patients between having a scan and not having a scan if they had a headache and what they showed was that people who had a scan there was some transient reassurance from the normal findings on the scan but if you followed them up by a yr or so between those who had the scan and those who didn’t anxiety levels were exactly the same between the two groups so the scan gives transient reassurance so I will tell them that that it’s not likely to change anything or find any surprises and any reassurance they benefit from it may not endure and that is probably because patients with headache want to be free of headache its usually not sufficient in the long term for patients to be told you don’t have a brain tumour it’s ok the usual reaction is that’s great news and relief but hang on a minute I’ve still got headaches fix them please

I: Do you think the problem is going to increase in the future

P: Definitely, MRI is becoming more available if the NHS is broken down and privatised and on has less ability to control patient pathways and their suitability for scanning or not then if it becomes a commercial incentive to scan more patients because the money flows with the scan and the report the commercial reorganisation of the NHS will undoubtedly increase if the NHS is not commercialised which has not occurred too much as yet in Scotland then we will still likely have an increase in all of this because MRI is becoming more available in clinical practice here an there its mere availability means it gets used more statistically it means you know more of these things will be discovered.

I: Have you noticed any differences with advances in imaging technology and higher field strength scanners

P: I’ve haven’t noticed any from 3T scanners on a personal level but in researching for the systematic review with other authors we tried looking at whether magnet strength altered the detection rate we couldn’t find anything in particular on magnet strength but we did find where higher resolution sequences were used 1mm slices from epilepsy protocols that kind of thing we found a greater detection of IF but its difficult to know whether that is due to the technology or is it the way the images are scrutinised by the radiologists having been acquired them in fine detail and then in reformatting them maybe spending longer viewing them so it’s not yet proven if the it’s the higher resolution images or the different way the radiologists view those images that uncovers the findings .

I: Do you hold any opinions and views regarding the management of patients with incidental findings?

P: Well once you have detected an IF they put you in a dilemma regarding how to manage it the important thing to address in clinical care especially when anticipating these things are going to be more frequently detected so for one of these conditions AVM’s there is a randomised control trial saying ok you have got an AVM you have never bled from your AVM many people have had seizures from them some have bled yours has never bled should we treat them or not so for part of my clinical practice for example very recently I saw a patient in clinic who had had an epileptic seizure and then had an unruptured AVM detected he entered this randomised control trial comparing a policy of treatment vs no treatment so I think that the whole IF phenomena will not only generate a clinical dilemma but because of the clinical dilemma they generate will generate research questions which then need research funding to answer them so that ultimately in 10-20 yrs time we may then know what the best thing to do with these IF so I think that is one of the many knock on ramifications regarding their detection. There was a randomised control trial regarding aneurysms should we coil them or should we leave them but they had great difficulty recruiting patients as currently does the AVM trial that I randomised someone into recently and there are various reasons for that but one of them is when a patient does realise they have something abnormal in their head and there is a risk of it bleeding one day they are not always as uncertain as we are about whether it should be treated or left alone its easy to feel the threat of the bleeding rather than the risk of complications of the treatment which are often in the short term much higher than the actual risk of bleeding

I: Have you encountered any other abnormalities with dilemma regarding their management

P: White matter disease are quite a common finding that recognising it as a disease requiring a specific treatment I don’t think is where medicine is at the moment there are research studies going on to say ok in people with white matter disease detected on MRI that looks like a vascular pattern does aspirin benefit these patients for example in preventing future vascular disease but simply the detection of leucariosis on an MRI or CT means one should tell patient to stop smoking and control their blood pressure but whether anyone actively does anything else regarding any treatment is unknown at the moment but that is the subject of research studies too

I: Are you experiencing any issues regarding patient outcome with incidental findings?

P: The only benefit would be patients who have had an incidentaloma detected who have come to the clinical service and elected to have treatment which precedes without complications and results in the successful treatment of the IF and preventing a bleed that would otherwise have occurred but because I don’t know which of those people would have had a bleed had we not treated them it is difficult to feel that benefit but all one can think I people who undergo treatment for one reason or another if it precedes safely and they have had an aneurysm coiled or an AVM treated and they got away without any complications of the procedure and the rest of their life goes fine then some of those people might have benefited because they would otherwise would have had a bleed but knowing who is who is difficult I haven’t ever had anybody bound into the room pleased to have found an IF, it is just intangible you don’t know who the people would have been who would have bled.

We should warn them of IF before the scans explain there is a 1 in 37 chance of finding something on an MRI basically they are playing roulette having an MRI. I try to impress upon patients who have headaches and who are determined to have a scan that’s the risk they are taking undertaking an MRI.

Participant 8

I: Incidental findings found within the brain are currently holding a lot of academic press at the moment, with patients being referred from clinical, research and private practice. I was wondering what your views were upon the topic.

P: Oh yes it is quite relevant, in my experience as a neurology trainee they would crop up regularly and depending on what it was it could cause quite a lot of problems certainly IF will require more investigations to verify what it is. I believe they are a real issue.

I: Could you describe how IF have impacted upon your workload

P: At the milder end of the spectrum IF for example arachnoid cysts which are cysts that have been there for a long time they aren’t that important I guess there are 2 steps in your thought process. There is deciding in your mind this IF isn’t important and doesn’t have any relation to the overall picture and doesn’t need any immediate action and then there’s communicating that to the patient which is a whole different entity and that this is just side effects of having a scan causing undue worry certainly on the odd occasion it is quite difficult to communicate that you often do it by letter but often you find it difficult to find a form of words which is not going to unduly worry somebody and then opening the doors to them wanting a whole load of unnecessary tests which they really don’t need –so I guess that is the milder end of the spectrum. And then you have more severe end where you find something that is incidental but however requires acting on as it is potentially serious. It is however incidental and is not what the patient was initially scanned for, the IF is unrelated to the clinical indication for the scan. Similarly it is quite difficult to explain to somebody why you are committing them on a result of that scan to more tests. I had a patient who was scanned for headache who was then found to have lots and lots of white matter disease. I thought ohh I didn’t ask about any inflammatory symptoms or anything suggestive of MS and what do I do now and then committing that person to a barrage of further tests and blood tests to look for inflammatory markers and a lumbar puncture. And a lumbar puncture is by no means easy on the patient and they could potentially end up with a headache but what I’m saying is it not a small thing. It hasn’t happened very often to me but I get certainly get it happening a couple of times a month if not more where something incidental has cropped up. More often than not it is an arachnoid cyst type thing.

I: Do you ever feel under pressure from patients to ask for further imaging to allay their fears

P: Yes interesting yes I do I tend to try not to, I lay it out on the table. I will say to them look if I really thought you needed a scan I would request one but I don’t but sometimes when you have somebody really anxious that doesn’t do and certainly in my experience I will end up agreeing to the scan. Also in my experience where the patient has functional problems and they have had a CT scan with the results sent back to the GP the GP then writes back to you and says look this patient is still having symptoms last time they had a CT scan but not an MRI. They know that the MRI scan is the gold standard and so they refer the patient back to you for more imaging so now to stop that happening you now ask for an MRI scan in the first instance because you know that now they are not going to be able to ask for anything else and I think that the functional category is the biggest category where I end up doing the scan so I know and everyone knows they have a normal scan the problem comes when the scan does not come back as completely normal.

I: In your experience how often do you feel the necessity to request further imaging or intervention?

P: Oh quite often, to further investigate or just to keep surveillance on those non-specific IF and of course to review meningioma’s.

I: Do you ever explain the risks of finding something incidental and unrelated to their symptoms

P: Yes I do talk about the risk of IF but only when I feel they really do not need a scan and they insist upon it and I’m trying to talk them out of having it. Most of the time no I don’t talk about it if I think they need a scan then I just go with that But certainly if people are trying to push me into having a scan I will be inclined to talk about IF the down side of having a scan but no I don’t discuss anything more specific you know I will broadly say there is always the chance we could pick something up that is totally unrelated to this I don’t quote figures, but I think it’s 1 in 37 people scanned will have an IF which is quite high

I: In your experience have you found any issues with managing patients found with IFs.

P: All the time yes the evidence based treatments for what we do is so limited it the approach tends to be what a reasonable body of people would do and which tends to be what people have done in the past or what I have done in the past. There will be discussions at the neuro-radiology meetings where lots of consultants with lots of experience whose combined experience will help guide the management process saying we wouldn’t take that any further or not but in terms of hard evidence based guidelines no there aren’t really any. Well for certain things like aneurysms there are certain criteria which is followed, if it’s in a certain location like the posterior circulatory system and of a certain size we would or wouldn’t do anything about but white matter changes it is very difficult to decide what to do after the usual blood tests it is better to be left untouched because what on earth in reality are we going to do we are not going to change anything they do not have any symptoms relevant to the actual problem so you are just going to wait until they do develop symptoms. With the coiling or clipping of aneurysms there are possible risks from the general anaesthetic, infection, infarct, bleeding, vasospasm and the fact that you have coiled somebody doesn’t mean you have 100% removed the aneurysm it might regrow requiring the procedure again with again all the risks attached. But also you have the personal issue of the patient knowing they have an aneurysm in their brain and that constant worry irrelevant to what you say that it might explode what will I do.

I: In your experience would you agree there are cost implications regarding IF for all involved

P: Yes yes there are costs in terms of our time talking with the patients allaying their fears which takes a huge amount of effort, researching the finding, research any evidence based managements, going to meetings to discuss the abnormality and the costs of the further testing further imaging further consultations and the psychological impact of the findings on the patient and the clinician the worry especially if the scan wasn’t clinically indicated in the first place. The patient too spends time off work having scans and consultations. We can also get continuous phone calls from worried patients it’s like a continuous stream of questions about this IF. Their consultations can take longer while we explain the IF and how we came to find it with them saying you didn’t explain there was a chance you could find something else. I find it a difficult conversation saying we have found something but we don’t need to do anything about it because that concept is so very difficult to get over to people and worse with people are anxious.

I: What are your personal opinions of IF?

P: I don’t like them I have enough to do without worrying about them as well. As I have gone through my training I think long and hard before I request a scan for which I do not have any clear justification and that’s chiefly done because it is good clinical practice and partly because I do not wish to uncover any IF and not knowing what to do with half of the IF if we get them. \it would be good to have more standardised ways of managing such things it does tend to be what a reasonable body of clinicians would do rather than an expert panel of judges there are no clinically proven evidence based guidelines on how to treat. As a junior perhaps not enough emphasis is placed upon the treatment of IF because as a junior you are told to be mindful of requesting CT brains due to the ionising radiation risk but you don’t often think about the chance of picking up something incidentally and it is definitely something we should think about more and we need more help and guidelines with evidence based practice to help manage these patients

I: Can you foresee the issue of IF growing in the future

P: For sure, as the MRI availability grows more people will be scanned. MRI has only been around for the last 20yrs with the pictures improving all the time also something new may come along in the next 10-20 yrs finding ever more to fill our time in the cause of our careers. The MRI scans are getting so sensitive they are picking things up earlier and earlier in the disease process we are still learning what the significance of these finding are. That combined with our ethical duty to confide these findings which we are unsure of what they mean if anything with the patient will cause a quandary so yer I believe the problem will grow.
